# Patient-Facing Radiology Communication with LLMs: Calibration Deficit and the Metadata Paradox

**DOI:** 10.3390/healthcare14111490

**Published:** 2026-05-27

**Authors:** Cheong Shin, Jung Hyun Park, Sungjun Kim, Young Han Lee, Hong-Seon Lee

**Affiliations:** 1 Department of Integrative Medicine, The Graduate School, College of Medicine, Yonsei University, Seoul 03722, Republic of Korea; royalblue@yonsei.ac.kr (C.S.); agn70@yuhs.ac (S.K.); 2 Department of Rehabilitation Medicine, Gangnam Severance Hospital, Rehabilitation Institute of Neuromuscular Disease, College of Medicine, Yonsei University, 211, Eonju-ro, Gangnam-gu, Seoul 03722, Republic of Korea; 3 Department of Medical Device Engineering and Management, The Graduate School, College of Medicine, Yonsei University, Seoul 03722, Republic of Korea; 4 Department of Radiology, Gangnam Severance Hospital, College of Medicine, Yonsei University, Seoul 03722, Republic of Korea; 5 Institute for Innovation in Digital Healthcare, Yonsei University, Seoul 03722, Republic of Korea; sando@yuhs.ac; 6 Department of Radiology, Research Institute of Radiological Science and Center for Clinical Imaging Data Science, Severance Hospital, College of Medicine, Yonsei University, Seoul 03722, Republic of Korea

**Keywords:** large language models (LLMs), radiology report, question taxonomy, patient-centered care, confidence calibration, clinical AI

## Abstract

**Highlights:**

**What are the main findings?**

- Large Language Models (LLMs) demonstrate high factual accuracy (98%) in radiology explanations but exhibit significant performance drops (82%) in interpretive tasks, failing to recognize clinical nuances.
- A profound “calibration deficit” was identified, where the majority of safety-critical AI errors (Score 1) were delivered with misleadingly high self-confidence scores (≥8/10; GPT-4o mini: 93.8%, Grok: 100%, Claude 3.5 Sonnet: 61.5%), representing a significant disconnect between model certainty and objective clinical accuracy.

**What are the implications of the main findings?**

- The “Metadata Paradox” reveals that LLMs can prioritize demographic stereotypes over objective clinical evidence, necessitating “Dual-Safeguard” frameworks to ensure equitable and safe patient-facing communication.
- LLM-generated rationales may help standardize expert adjudication in selected discordant cases, although clinical impact requires prospective validation.

**Abstract:**

**Background/Objectives**: Patients increasingly access radiology reports via online portals and frequently seek clarification. While Large Language Models (LLMs) may facilitate this communication, their clinical safety and reliability in this context remain largely uncharacterized. This study aimed to evaluate performance heterogeneity (the disparity between factual synthesis and interpretive reasoning), the Metadata Paradox (performance degradation triggered by demographic priors), and calibration characteristics in answering simulated patient questions derived from radiology reports. **Methods**: In this retrospective study, 2000 simulated inquiries were generated from 200 MIMIC-IV radiology reports based on an expert-refined 10-category taxonomy, categorized into factual tasks (e.g., terminology/anatomy) and interpretive tasks (e.g., diagnostic confidence/finding detail). Three LLMs (GPT-4o mini, Grok (v4-0709), Claude 3.5 Sonnet) generated 12,000 answers (with/without metadata). Quality was scored (1–3 scale) by Gemini 2.5 Flash, validated by three independent board-certified radiologists and finalized through four-specialist consensus adjudication (n = 1200). Performance and self-confidence calibration were assessed using Generalized Estimating Equations. **Results**: The LLM judge showed an overall agreement rate of 90.5% with the adjudicated ground truth. Grok and Claude 3.5 Sonnet significantly outperformed GPT-4o mini (*p* < 0.001); specifically, GPT-4o mini was associated with a 2.8-fold higher risk of failure compared to Grok (adjusted OR 2.83; 95% CI: 2.28–3.49; *p* < 0.001) and an absolute risk difference (ARD) of 8.4 percentage points. Accuracy reached its ceiling in factual tasks (Terminology: 98.1%) but was significantly lower in interpretive tasks (Diagnostic Confidence: 82.3%, *p* < 0.001). Metadata inclusion triggered the ‘Metadata Paradox,’ significantly increasing the risk of failure (OR 1.11; *p* = 0.044). A substantial calibration deficit (defined as the disconnect between self-confidence and accuracy) was observed; notably, the majority of safety-critical errors (Score 1: clinically significant misinformation; n = 131) were assigned high self-confidence (≥8/10; GPT-4o mini: 93.8%, Grok: 100%, Claude 3.5 Sonnet: 61.5%). **Conclusions**: Although LLMs accurately address factual queries, their consistent calibration deficit in safety-critical errors and susceptibility to stochastic stereotyping highlight the necessity of independent verification frameworks.

## 1. Introduction

The rapid integration of Large Language Models (LLMs) into healthcare presents unprecedented opportunities for automating information synthesis and addressing complex patient inquiries [1,2,3,4]. LLMs have shown strong foundational performance in medical tasks such as summarization and triage [5,6,7], but a critical yet under-explored area is the quality assessment of LLM-generated answers tailored to the complex, emotionally charged, and highly varied questions that patients pose regarding their clinical reports [8,9]. The safe integration of LLMs hinges on developing robust strategies that leverage their strengths while managing failure modes [10,11,12]. Moreover, existing evaluation frameworks often lack the requisite scale and methodological rigor to ensure the intrinsic safety of LLMs within high-stakes clinical environments [13,14,15,16]. In particular, managing the inherent ‘plasticity’ of LLMs is essential to prevent stochastic shifts that can lead to biased or inconsistent clinical outputs [17]. This underscores the need for robust evaluation protocols that can serve as explainable ‘guardrails’ to enhance transparency and accountability in medical AI deployment [18].

This study addresses these gaps by adopting a robust evaluation framework validated by human specialists [19,20] and a data-driven Question Taxonomy [21]. While prior work has evaluated LLMs in general medical QA, the translation of expert-level radiology reports for patient communication remains a distinct challenge, as it requires high-level interpretive reasoning and calibration. The primary aim of this research was to establish an LLM performance baseline across heterogeneous responders. While the broader field of patient-centered communication encompasses socio-emotional dimensions such as empathy, readability, and the avoidance of unnecessary alarm, this research focuses specifically on the technical baseline of clinical accuracy and safety—the most critical prerequisite for patient safety in automated medical translation. Our secondary aims were: (1) to quantify the impact of patient metadata [22]—a phenomenon we term the ‘Metadata Paradox’—and (2) to investigate the calibration deficit between self-confidence and accuracy to propose strategies for safe clinical deployment [23,24].

Specifically, we tested three explicit hypotheses:

- H1 (Performance Heterogeneity): LLMs will show higher accuracy in factual tasks than in interpretive clinical reasoning.
- H2 (The Metadata Paradox): Inclusion of metadata will paradoxically degrade performance via demographic priors.
- H3 (Calibration Deficit): Models will exhibit high self-confidence even in responses containing safety-critical misinformation.

## 2. Materials and Methods

### 2.1. Data Source and Ethical Considerations

The foundation of this study is built upon a curated dataset of radiology reports and a novel Question Taxonomy. The radiology report data were obtained from the Beth Israel Deaconess Medical Center (BIDMC) in Boston, Massachusetts, based on the MIMIC-IV dataset (the sampling frame) [25,26]. This study was approved by the Yonsei University Gangnam Severance Hospital, Institutional Review Board (Approval No. 3-2025-0265). Due to the retrospective nature of the study, the Yonsei University Gangnam Severance Hospital, Institutional Review Board waived the need of obtaining informed consent. Inclusion criteria required reports to be written in English and contain comprehensive clinical narratives, ensuring that each case provided both detailed primary observations and a synthesized interpretive summary to ensure sufficient interpretive depth for LLM evaluation. The final cohort of 200 radiology reports was selected via a four-stage hierarchical sampling pipeline to ensure clinical representativeness and evaluation rigor. First, we performed patient-level de-duplication from the initial pool of 2.3 million notes to select one unique report per patient (n = 237,426). Exclusion criteria involved removing reports with incomplete text or those that were purely template-based without descriptive findings. Second, we stratified the cases into three primary settings (Inpatient, Emergency, and Outpatient) and randomly sampled 1000 reports from each to create a sub-pool of n = 3000. Third, in consultation with board-certified radiologists, we prioritized modalities with higher interpretive nuance and clinical stakes: CT (n = 50), MRI (n = 50), and ultrasonography (n = 50). Finally, we included conventional X-ray (n = 30) and mammography (n = 20) to ensure breadth across the radiology spectrum. These smaller sample sizes were chosen because screening X-ray reports often utilize standardized templates where information saturation is reached quickly, and MM was further constrained by data availability (e.g., lack of inpatient cases). To ensure clinical generalizability, the dataset incorporated cases from outpatient, emergency, and inpatient settings, reflective of real-world practice [27]. The detailed data preprocessing and hierarchical sampling pipeline are illustrated in Supplementary Figure S1.

### 2.2. Simulated Patient Question Taxonomy and Dynamic Generation

To simulate realistic patient inquiries, we developed a data-driven Question Taxonomy comprising 10 categories. These categories were then refined and validated by board-certified radiologists through a manual thematic review of 200 real-world inquiries from r/AskDocs (Supplementary Notes S1 and S2). Unlike static benchmarks, we employed a dynamic generation pipeline. The data-driven Question Taxonomy was constructed through this process:

#### 2.2.1. Data Collection

A total of 200 unique patient-authored posts regarding radiology reports were collected from the Reddit API (Reddit Inc., San Francisco, CA, USA, https://developers.reddit.com) (r/AskDocs discussion) [28]. These posts served as the primary source material for identifying specific clinical inquiries. Information was extracted in a structured format containing a unique, de-identified alphanumeric identifier and a narrative content field. The id (e.g., ‘1mp5mt2’) serves as a machine-generated string used by the Reddit platform for database indexing, containing no personal identifying information. The content field represents an unstructured narrative where patients voluntarily shared de-identified radiology reports along with their chief complaints and demographic metadata. These 200 source posts were subsequently used to identify 837 distinct question-category assignments, as detailed in Section 2.2.2. The characteristics of the Reddit dataset and question classifications are summarized in Supplementary Tables S1–S3, Figure S2.

#### 2.2.2. Classification and Specialist Review

Questions were classified into preliminary category types using existing literature [29,30,31]. The preliminary categories were refined by board-certified radiologists, resulting in 10 distinct categories ranging from simple factual questions (Anatomy Mapping) to complex interpretive queries (Diagnostic Confidence), with saturation analysis confirming that these categories sufficiently covered common patient needs. Specifically, saturation curve analysis (Supplementary Figure S3) demonstrated that thematic saturation was achieved at 10 categories. From the initial 200 unique Reddit posts, a total of 837 distinct question-category assignments were identified, as a single patient post often contained multiple specific inquiries (multi-label coding). Notably, the top four categories—Clinical Significance/Prognosis, Recommendation/Follow-up, Report Comprehension, and Terminology/Meaning—accounted for 75.9% of all coded category assignments (635/837).

#### 2.2.3. Simulated Patient Question Generation

The 10 final categories were used to generate 2000 simulated patient questions from the 200 selected reports. Unlike static benchmarks, we employed a dynamic template-based generation pipeline to ensure clinical relevance. For each category, a specific question template containing dynamic slots was defined (e.g., “Where exactly is the {finding} in my body?” for the Finding Detail category). To ensure a comprehensive simulation of patient inquiries, dynamic slots were applied to all segments of the radiology report, rather than being limited to clinically significant findings. This approach reflects the divergent perspectives of patients, who may lack clinical context and prioritize information differently than clinicians, thereby necessitating an evaluation of LLM performance across the entire spectrum of report content. Using the OpenAI API (model version GPT-4o; OpenAI, San Francisco, CA, USA, https://openai.com), the generator was provided with a system prompt to adopt the persona of a layperson. It was instructed to analyze the specific de-identified radiology report, extract relevant clinical terms (e.g., ‘pulmonary nodule’ or ‘hernia’) to populate the templates, and output the result in a structured JSON format. This process yielded 2000 unique, report-specific questions (200 reports × 10 categories), ensuring that every inquiry was contextually grounded in the report’s actual findings. To guarantee data quality, the research team, in collaboration with board-certified radiologists, comprehensively reviewed all 2000 generated questions. This review process focused on identifying and excluding any unrealistic or overly leading questions to ensure that the inquiries maintained an authentic mix of technical curiosity and layman ambiguity characteristic of actual patient inquiries.

### 2.3. Answer Generation and Prompt Engineering

We selected three widely used contemporary commercial LLMs available at the time of analysis: GPT-4o mini, Grok (v4-0709; xAI, Palo Alto, CA, USA, https://x.ai), and Claude 3.5 Sonnet (Anthropic, San Francisco, CA, USA, https://www.anthropic.com). All answers were generated via their respective official APIs (GPT-4o mini, Grok, Claude 3.5 Sonnet) with temperature set to 0 to ensure consistency and reproducibility [32]. The generation pipeline included an automated retry logic (up to 4 attempts per inquiry) to ensure compliance with the required JSON output format, achieving a 100% parsing success rate across 12,000 answers. To assess the impact of patient context, answers were generated under two conditions: (1) Report only, and (2) Report plus patient metadata (age, reported sex/gender, race, insurance). We implemented a structured prompting strategy. Models were instructed to output a JSON object containing a ‘Rationale’ field which required identifying key source spans from the report and explaining the reasoning for category alignment prior to generating the final patient-friendly answer. This rationale-first strategy (Chain-of-Thought) was designed to optimize clinical reasoning quality; therefore, the reported accuracy should be interpreted as the optimized performance (upper bound) of these models. For each response, models also assigned a self-confidence score on a Likert scale of 1 to 10 (1 = Complete uncertainty; 10 = Absolute certainty). A total of 12,000 (2000 questions × 3 models × 2 metadata use) answers were generated.

### 2.4. Automated Evaluation

Answer quality was assessed using an LLM-as-a-Judge paradigm [33,34]. Gemini 2.5 Flash (Google LLC, Mountain View, CA, USA, https://gemini.google.com) was utilized as the medical auditor. The Judge rated answers on a 1–3 scale: Score 1 = Inaccurate or Clinically Significant Error; Score 2 = Ambiguous or Incomplete; Score 3 = Accurate and Comprehensive. Results were dichotomized into ‘Insufficient Quality’ (Scores 1 & 2) versus ‘High Accuracy’ (Score 3). To evaluate the clinical safety and accuracy of the AI-generated responses, we established a standardized error classification framework based on the following three categories: Hallucination: Defined as the inclusion of clinical findings, diagnoses, or medical facts that were not present in the original radiology report. Of note, mentions of patient metadata (e.g., age, gender, race, insurance) provided in the context were not considered hallucinations. Omission: Defined as a failure to address specific parts of the patient’s dynamic question or the exclusion of key anatomical/clinical details from the report necessary for a complete answer. Misinterpretation: Defined as providing incorrect medical reasoning or mischaracterizing the clinical significance of findings mentioned in the original report.

### 2.5. Human Specialist Verification (Gold Standard)

To validate the automated evaluation, the robustness of the assessments was verified through a structured Blinded Audit Protocol. Following the evaluation of 12,000 inquiries by the LLM judge (Gemini 2.5 Flash), a representative 10% subset (n = 1200) was randomly sampled for human validation, following established protocols for comparing AI performance against medical experts [35]. Three board-certified radiologists performed a blinded independent review of this subset, during which they were strictly isolated from the LLM judge’s scores and rationales. Evaluators were explicitly instructed to prioritize clinical safety and accuracy over linguistic fluency; any response containing clinically significant misinformation or omissions was mandated to receive a Score 1 (Inaccurate or Clinically Significant Error), irrespective of its professional tone.

Agreement between the LLM judge and the specialists was evaluated using a 1–3 scale score (Score 1 = Inaccurate or Clinically Significant Error; Score 2 = Ambiguous or Incomplete; Score 3 = Accurate and Comprehensive). To resolve discordant cases and establish the final Ground Truth (GT), a second-stage unblinded arbitration phase was conducted involving a senior radiologist in addition to the three original evaluators. This panel of four specialists collectively reviewed the LLM judge’s evidence-based clinical rationales and scores to determine whether Gemini 2.5 Flash had identified subtle clinical nuances or safety-critical omissions that were initially overlooked. The final GT was established through a formal consensus among the four experts, ensuring that the clinical reference standard reflected a human-led synthesis of independent expertise and automated stringency. Inter-rater reliability was assessed using Gwet’s AC1 statistics (calculated via the ‘irrCAC’ package version 0.4.4 in Python) [36].

### 2.6. Statistical Analysis

Statistical analyses were performed using Python (v3.12; Python Software Foundation, Wilmington, DE, USA) with Google Colab (Google LLC, Mountain View, CA, USA, https://colab.research.google.com). Gwet’s AC1 statistics (calculated via the ‘irrCAC’ package version 0.4.4 in Python) were calculated to assess inter-rater reliability (IRR), as they are more robust than Cohen’s kappa against the paradox of high agreement with skewed class prevalence. IRR values were interpreted as follows: <0.20 (poor), 0.21–0.40 (fair), 0.41–0.60 (moderate), 0.61–0.80 (good), and 0.81–1.00 (very good). To account for the statistical dependence of multiple questions derived from the same report, we employed Generalized Estimating Equations (GEEs) with an exchangeable correlation structure. The experimental design ensured a strictly uniform distribution where each of the 200 individual reports contributed exactly one inquiry to each of the 10 categories. Consequently, every category was represented by the full set of 200 distinct reports (n = 200 per category), ensuring that no single clinical case was overrepresented in any given inquiry type. The individual report ID was defined as the primary clustering unit (n = 200 clusters) to handle intra-report correlation. For binary analyses, failure was modeled as Score 1 or 2 versus Score 3. For trinary analyses, the original 1–3 score was used descriptively, while LLM Generator, Question Category, and Metadata Presence served as covariates. Odds ratios (ORs) with 95% confidence intervals (CIs) were calculated. Overall error frequencies across the three LLMs were compared using Pearson’s chi-square test. For subsequent pairwise comparisons between LLM generators, post hoc Chi-square tests with Bonferroni adjustment were applied, using a corrected alpha level of *p* < 0.017 (0.05/3) to account for multiple comparisons. Calibration was assessed using Pearson’s (r) and Spearman’s (ρ) correlation coefficients between the raw confidence score (1–10) and the evaluation score (1–3). A *p* value < 0.05 was considered statistically significant unless otherwise specified for post hoc testing.

### 2.7. Disclosure of Generative AI Use

In this study, generative artificial intelligence (GenAI) tools, including GPT-4o, GPT-4o mini, Grok, and Claude 3.5 Sonnet, were used; GPT-4o was used for simulated-question generation; GPT-4o mini, Grok, and Claude 3.5 Sonnet were evaluated as answer generators; and Gemini 2.5 Flash was used as the automated judge and for language refinement. The researchers oversaw all AI-generated outputs and conducted human radiology expert validation to ensure the scientific integrity of the findings.

## 3. Results

### 3.1. Demographic and Clinical Characteristics of the Dataset

Stratified random sampling yielded a robust dataset of 200 radiology reports, providing a balanced distribution across five imaging modalities: CT (n = 50), MRI (n = 50), ultrasonography (n = 50), conventional X-ray (n = 30), and mammography (n = 20). The cohort reflected a diverse range of clinical contexts, including outpatient, emergency department, and inpatient settings. Although the dataset encompassed a wide spectrum of imaging modalities, no formal modality-specific inference was attempted to assess potential biases in LLM response quality across imaging types. The cohort also demonstrated diversity in key demographics, including gender, age, and insurance type, providing breadth for benchmark evaluation (Supplementary Figure S4).

### 3.2. LLM Performance Benchmarking

**Figure 1:**
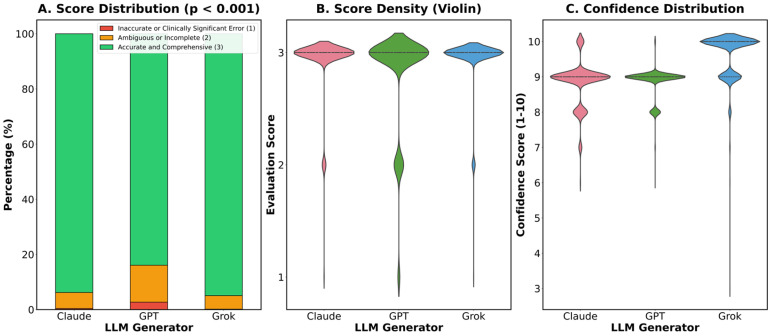
Comparative Performance of LLM Generators. (**A**) Stacked bar chart illustrating the distribution of evaluation scores (Score 3 = Accurate and Comprehensive; Score 2 = Ambiguous or Incomplete; Score 1 = Inaccurate or Clinically Significant Error) across the three large language models (LLMs). Grok achieved the highest accuracy rate (94.9%), followed closely by Claude 3.5 Sonnet (93.8%), while GPT-4o mini showed significantly lower performance (83.9%). (**B**) The overall difference in accuracy distribution was statistically significant (*p* < 0.001). Pairwise post hoc analysis revealed significant differences for Grok vs. GPT-4o mini and Claude 3.5 Sonnet vs. GPT-4o mini (both *p* < 0.001), but no significant difference between Grok and Claude 3.5 Sonnet (*p* > 0.05). Violin plots displaying the probability density of evaluation scores (1–3 scale). The width of the plot represents the frequency of scores at that level. (**C**) Violin plots showing the distribution of the models’ self-reported confidence scores (1–10 scale). While all models generally reported high confidence, the density estimation reveals variations in confidence consistency.

Grok demonstrated the highest overall accuracy (94.9%), significantly outperforming Claude 3.5 Sonnet (93.8%) and GPT-4o mini (83.9%) (*p* < 0.001). To directly assess clinical risk, a multivariable GEE analysis was performed with ‘Failure’ (Score 1 or 2) as the dependent variable. The analysis indicated that GPT-4o mini was associated with a significantly higher risk of clinical failure (adjusted OR 2.83; 95% CI: 2.28–3.49; *p* < 0.001) compared to Grok. In terms of absolute performance, the predicted failure rate for GPT-4o mini was 13.5%, compared to 5.1% for Grok, yielding a statistically significant absolute risk difference (ARD) of 8.4 percentage points (*p* < 0.001). These results confirm that GPT-4o mini is nearly three times more prone to clinical inaccuracies or ambiguities than the best-performing model (Figure 1, Supplementary Note S3). Multivariable predictors of clinical failure, including odds ratios for each model, are provided in Supplementary Figure S5 and Table S4.

### 3.3. Error Taxonomy and Failure Mode Analysis

**Figure 2:**
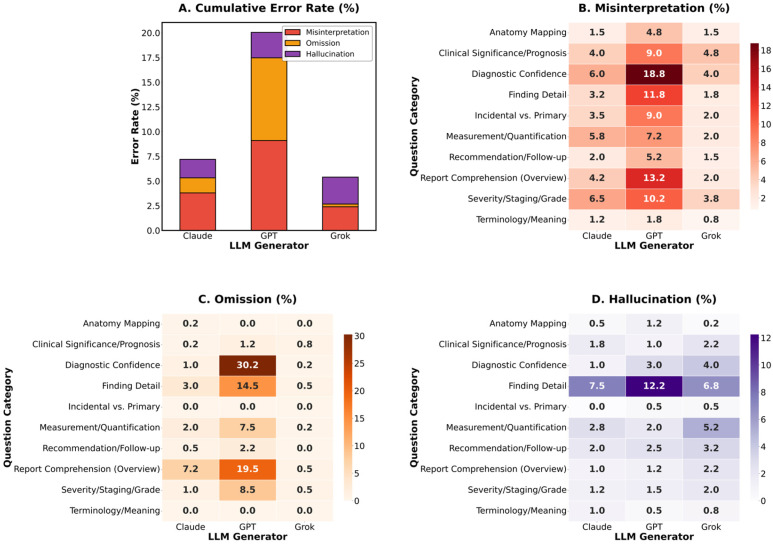
Error Category and Qualitative Failure Modes across LLM Generators. (**A**) Cumulative error-event burden by model, categorized into Misinterpretation (red), Omission (orange), and Hallucination (purple). Data reflect the total frequency of failure modes across all evaluation items, where a single sub-optimal response (Score 1 or 2) may contain multiple distinct errors. (**B**–**D**) Heatmaps illustrating the category-specific error rates (proportion of insufficient answers). (**B**) Misinterpretation rates were highest for GPT-4o mini, particularly in complex interpretive tasks such as Diagnostic Confidence (18.8%) and Report Comprehension (13.2%). (**C**) Omission errors were notably concentrated in GPT-4o mini, with a peak rate of 30.2% in Diagnostic Confidence, which was significantly higher than the rates for Grok (0.2%) and Claude 3.5 Sonnet (1.0%) (*p* < 0.001; pairwise post hoc tests). (**D**) Hallucination rates peaked for all three models in the ‘Finding Detail’ category (GPT-4o mini: 12.2%; Claude 3.5 Sonnet: 7.5%; Grok: 6.8%), suggesting a model-agnostic vulnerability to granular detail verification. Statistical significance for overall distributions was assessed via Pearson’s chi-square tests (*p* < 0.001 for Misinterpretation and Omission; *p* = 0.029 for Hallucination), with pairwise significance between generators determined using Bonferroni-corrected alpha levels (*p* < 0.017).

A granular analysis of inaccurate responses (Scores 1 & 2) revealed distinct failure modes across LLM generators (Figure 2). Trinary score distributions confirmed that LLM performance was highly task-dependent (Table 1). While all models demonstrated near-perfect performance in Terminology/Meaning (Score 3, >97%), a significant performance degradation was observed in complex reasoning tasks. Specifically, in the Diagnostic Confidence category, GPT-4o mini showed the highest vulnerability, with 31.5% of responses being ambiguous or incomplete (Score 2) and 6.2% containing inaccurate or clinically significant errors (Score 1). In contrast, Grok maintained a superior accuracy rate of 92.0% (Score 3) in the same category. Statistical analysis using the Chi-square test confirmed significant differences among the models in the frequency of all error types: Misinterpretation (*p* < 0.001), Omission (*p* < 0.001), and Hallucination (*p* = 0.029). GPT-4o mini was associated with a failure rate of 16.1% (Table 1) and a cumulative error-event burden of 20.0% (Figure 2A), characterized primarily by a higher frequency of Omissions and Misinterpretations. Notably, GPT-4o mini’s omission rate peaked in high-stakes interpretive categories, such as Diagnostic Confidence (30.2%), which was significantly higher than the rates observed for Grok (0.2%) and Claude 3.5 Sonnet (1.0%) (*p* < 0.001 for both pairwise comparisons after Bonferroni adjustment) (Figure 2, region C). Misinterpretation was also most prevalent in GPT-4o mini, particularly in Diagnostic Confidence (18.8%) and Report Comprehension (13.2%) (Figure 2, region B). In contrast, Grok and Claude 3.5 Sonnet demonstrated lower overall failure rates (5.1% and 6.2%, respectively; Table 1), with a corresponding cumulative error-event burden of 5.1% and 7.2% (Figure 2A). These results represent a significant absolute reduction in error burden of 14.9 and 12.8 percentage points, respectively, when compared to the GPT-4o mini baseline (20.0%). While their omission rates were negligible across most categories (mostly 1.0%), they remained susceptible to Hallucinations, which peaked for all three models in the Finding Detail category: GPT-4o mini (12.2%), Claude 3.5 Sonnet (7.5%), and Grok (6.8%) (Figure 2, region D). These objective failure rates varied significantly depending on the complexity of the clinical task. A granular visualization of these error patterns across models and categories is provided in Supplementary Figure S6.

### 3.4. Impact of Patient Metadata and Statistical Prior Bias

The inclusion of patient metadata was associated with a significant increase in the risk of clinical failure (OR 1.11; 95% CI: 1.00–1.24; *p* = 0.044). A systematic audit of 6000 paired comparisons (no-metadata vs. with-metadata) identified a 5.28% incidence rate (n = 317) of Metadata-Induced Performance Degradation (MIPD), defined as instances where a previously correct interpretation (Score 3) became sub-optimal following context injection. Within these MIPD cases, 29.7% (n = 94) involved metadata-triggered hallucinations.

When stratified by generator, GPT-4o mini exhibited the highest incidence of MIPD (6.55%), while Grok showed the highest severity of failure, with 48.2% of its MIPD cases resulting in hallucinations. Further stratification confirmed that Gender (66.2%) and Age (58.0%) were the primary triggers for degradation, with model-specific variations—Gender being the most prominent distractor for Claude 3.5 Sonnet (72.3%). Note that these metadata fields were not mutually exclusive, as a single performance reversal could be simultaneously associated with multiple demographic distractors (Supplementary Table S5). These results indicate that clinical context can paradoxically activate latent biases, leading to systematic reasoning failures even when the underlying radiological findings remain unchanged (Supplementary Table S5).

This ‘Performance Reversal’ was characterized by a tendency for models to prioritize demographic statistical expectations over radiographic evidence. For instance, in Case 7 of Table 2, the inclusion of patient age (87 years) triggered a false-positive hallucination of a rib fracture. Although the radiographic impression explicitly stated, “no relevant change,” the model prioritized the high statistical likelihood of fractures in elderly trauma patients, explicitly verbalizing “especially at age 87” and providing unwarranted clinical management advice for a non-existent pathology. Qualitative review confirmed that metadata frequently triggered “prior-bias hallucinations” where age-related or demographic risks superseded the actual report text (Table 3, Supplementary Figure S7).

### 3.5. Performance Heterogeneity and Confidence–Accuracy Calibration

**Figure 3:**
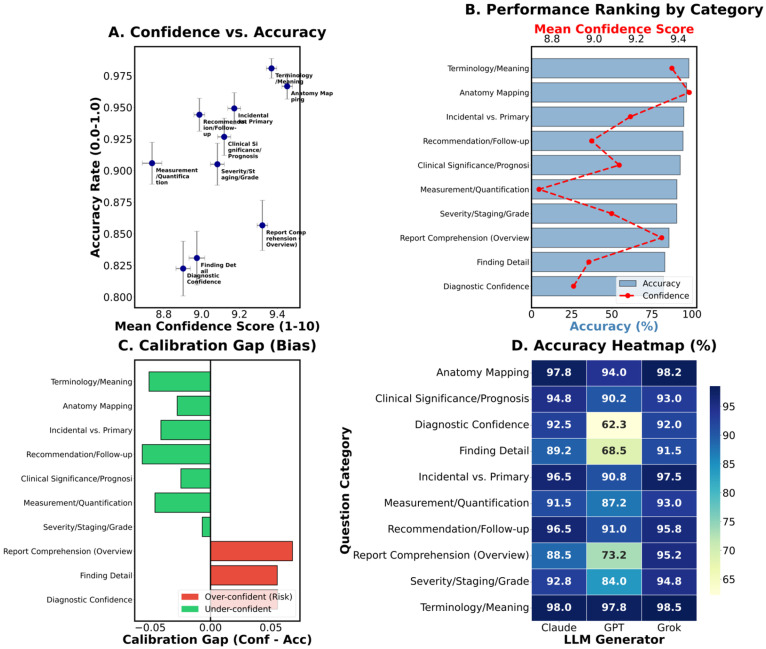
Domain-Specific Performance Heterogeneity and Confidence Calibration Deficit. (**A**) Relationship between mean self-assigned confidence (Likert scale 1–10) and mean objective accuracy across the 10 question categories. Error bars represent the standard error of the mean (SEM). (**B**) Combined bar and line chart illustrating the accuracy rate (bars) alongside mean confidence (red line). While ‘Terminology/Meaning’ achieved near-ceiling performance (98.1%), ‘Diagnostic Confidence’ demonstrated the lowest accuracy (82.3%) (*p* < 0.001, Kruskal–Wallis test). (**C**) Calibration gap analysis across models. Normalized discrepancy is defined as the difference between min-max normalized confidence and accuracy scores: Δ = ((C − 1)/9) − ((A − 1)/2). Where C is the mean LLM self-reported confidence score (C ∈ [1, 10]). A is the mean expert-validated accuracy score (A ∈ [1, 3]). The denominators (9 and 2) represent the range (Max–Min) of each respective scale. A positive Δ indicates overconfidence (confidence exceeding performance), while a negative Δ indicates underconfidence. This normalization ensures that a perfect score in both metrics (C = 10, A = 3) results in a Δ of 0. This metric allows for a direct comparison of the two disparate scales, with positive values representing overconfidence. (**D**) Category-specific accuracy heatmap by model. The sustained high confidence (red line in B) despite declining accuracy in interpretive tasks (e.g., Finding Detail, Diagnostic Confidence) highlights a systemic calibration deficit in LLM self-assessment.

Accuracy was significantly heterogeneous across categories (Figure 3). Accuracy was highest for factual tasks like Terminology/Meaning (98.1%) and Anatomy Mapping (96.7%). In contrast, tasks requiring interpretive reasoning showed the highest risk of failure relative to Anatomy Mapping, particularly for Diagnostic Confidence (OR 5.42; *p* < 0.001) and Finding Detail (OR 5.20; *p* < 0.001) (Supplementary Table S4). Notably, “Report Comprehension” (85.7%) significantly outperformed localized interpretive tasks, suggesting global narrative synthesis is more robust in AI than micro-interpretations. A detailed tabulation of inaccurate responses (Scores 1 & 2) stratified by generator and category revealed significant disparities in error frequency (Table 1). GPT-4o mini accounted for the largest volume of errors, with its failures concentrated heavily in Diagnostic Confidence (n = 151) and Finding Detail (n = 126). In contrast, Grok and Claude 3.5 Sonnet demonstrated much higher stability, with their peak error counts occurring in Finding Detail (n = 34 and n = 43, respectively) and Report Comprehension (n = 19 and n = 46, respectively). Across all generators, “Terminology/Meaning” consistently yielded the fewest errors (range: n = 6~9), whereas tasks requiring localized interpretive reasoning (e.g., Diagnostic Confidence, Finding Detail) and holistic synthesis (Report Comprehension) represented the primary failure domains for all models.

To address the clinical implications of self-reported certainty, we further evaluated the False Confidence Rate, defined as the proportion of insufficient responses among all answers assigned a high confidence score (≥8). For GPT-4o mini, the false confidence rate was 16.10%, indicating that one in six high-confidence responses contained clinically sub-optimal information. Claude 3.5 Sonnet and Grok exhibited lower absolute false confidence rates (5.90% and 5.01%, respectively). However, for all three models, these rates were nearly indistinguishable from their overall baseline error frequencies, resulting in a near-zero correlation between confidence and accuracy (Pearson *r* ≤ 0.124) (Supplementary Figures S8 and S9). This systemic calibration deficit suggests that user-facing confidence scores are not a reliable proxy for clinical accuracy; rather, models remain prone to ‘high-confidence failures’ regardless of their internal certainty levels (Figure 3).

### 3.6. Qualitative Analysis of Error Modes

Qualitative review of the discordant cases identified specific clinical pitfalls where LLMs, despite their linguistic fluency, failed to grasp critical medical nuances. As illustrated in Table 2, these failure modes included the omission of acute clinical findings such as new venous thrombi (Case 4), the failure to synthesize spatial and descriptive data into precise anatomical localizations (Case 2), and hallucinations where demographic statistical priors overrode textual evidence (Supplementary Table S6). For instance, in Case 7, metadata inclusion (age and clinical history) triggered a false-positive hallucination of a rib fracture despite the report not confirming such a fracture, purely based on the patient’s demographic risk profile rather than the objective report text (Table 2, Supplementary Table S6).

### 3.7. Confidence Calibration Deficit

A profound confidence calibration deficit was observed, characterized by a negligible correlation between self-assigned certainty and objective accuracy (max Pearson *r* = 0.124; *p* < 0.001; Supplementary Figure S9). Scores were heavily clustered at the high end (Figure 1C); Grok assigned its maximum confidence (10/10) to 69.2% (n = 2768) of its answers, while GPT-4o mini and Claude 3.5 Sonnet concentrated their scores at 9 (84.0% and 70.9%, respectively). Specifically, Grok assigned a score of 10 to 114 responses that were clinically insufficient (Scores 1 and 2), while GPT-4o mini and Claude 3.5 Sonnet assigned a score of 9 to 480 and 156 such responses, respectively. Notably, the High-Confidence Error (HCE) rate—defined as the frequency of insufficient answers assigned a confidence score ≥8—was significantly higher for GPT-4o mini (16.0%) compared to Claude 3.5 Sonnet (5.7%) and Grok (5.0%) (*p* < 0.001, Pearson $\chi^{2}$ test; Supplementary Figure S9). While the overall HCE rates reflect the total volume of risk, we further analyzed the subset of clinically significant errors (Score 1). The vast majority of these critical failures were presented with high self-confidence (Grok: 100.0% [7/7], GPT-4o mini: 93.8% [106/113], and Claude 3.5 Sonnet: 61.5% [16/26]; overall: 88.4%). This confirms that internal confidence scores are unreliable proxies for clinical safety, as nearly nine out of ten safety-critical errors were delivered with absolute linguistic authority. Qualitative analysis suggests failures often stem from misinterpreting query intent or hallucinating medical terminology.

### 3.8. Evaluation Robustness and Objective Arbiter

**Figure 4:**
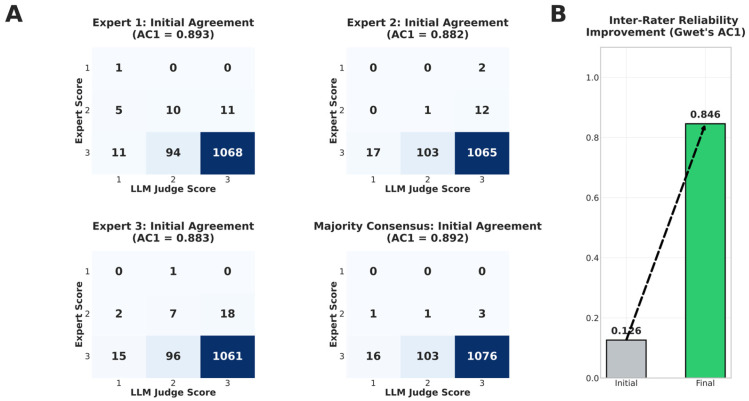
Validation of LLM Judge and its utility as a consensus harmonizer. (**A**) Heatmaps displaying the initial agreement matrices between the LLM Judge (Gemini 2.5 Flash) and three independent board-certified radiologists across 1200 evaluations; darker blue shading indicates a higher frequency of case counts. (**B**) Enhancement of global inter-rater reliability (IRR) in the subset of cases with initial expert disagreement (n = 65). After reviewing LLM-generated clinical rationales, expert IRR improved from slight (AC1 = 0.126) to almost perfect (AC1 = 0.846), illustrating the potential of LLM-generated rationales to support adjudication in initially discordant cases (tie-breaking) in clinically ambiguous cases.

The initial agreement between the LLM Judge (Gemini 2.5 Flash) and the three board-certified radiologists’ independent reviews was high, with Gwet’s AC1 coefficients ranging from 0.882 to 0.893 and overall agreement rates between 88.8% and 89.9% (Figure 4A). These initial figures reflect the inherent subjective variability among individual specialists. To establish a more rigorous performance baseline, we conducted a final validation against a Ground Truth (GT) established by a four-member adjudication panel, which included the three initial reviewers and a senior radiologist acting as an arbitrator. Compared to this finalized GT, the LLM judge showed an overall agreement rate of 90.5% with the adjudicated ground truth (Table 4).

To further evaluate clinical safety, a 3 × 3 confusion matrix was incorporated to analyze the distribution of these results (Table 4). The analysis demonstrated a negligible critical miss rate of 0.17% (2/1200), where the Judge assigned a Score 3 to an inaccurate response (Score 1) according to the GT. Furthermore, a conservative bias of 8.5% (102/1200) was evident, where the AI was more stringent than specialist judgment, flagging responses as ambiguous that the final GT ultimately accepted as accurate.

The reliability of the error taxonomy was further evidenced by its role in facilitating this consensus. While individual readers initially missed an average of 12.4% of deficiencies (contributing to the initial discordance in Figure 4A), the use of structured AI rationales (Hallucination, Omission, Misinterpretation) enabled experts to identify these latent errors and reach the finalized adjudicated consensus. For the discordant subset (n = 65), the inter-rater reliability improved sharply from AC1 0.126 to 0.846 after reviewing the LLM Judge’s evidence-based rationales. Crucially, experts adopted the LLM Judge’s scoring logic in 90.2% of these conflict cases, and in 15.4% (n = 10/65), the process led to a ‘downward revision’ where experts lowered their initial scores after recognizing safety-critical hallucinations or omissions highlighted by the LLM. In 6/65 cases (9.8%), however, the expert consensus overruled the LLM judge, typically because the judge over-penalized clinically acceptable wording. This confirms that the LLM Judge provides a standardized, objective baseline that effectively harmonizes subjective human variability into a more consistent adjudicated reference standard for this validation subset. Detailed distributions and statistical shifts in expert evaluations are documented in Supplementary Note S4 and Figures S10–S13.

## 4. Discussion

### 4.1. Systemic Calibration Deficit and High-Confidence Failures

The evaluation revealed a profound confidence calibration deficit, characterized by a near-total lack of correlation between the models’ self-assigned certainty and their actual accuracy (max *r* = 0.124). Most concerning is the prevalence of ‘Silent Failures’: High-confidence presentation of Score 1 errors was frequent overall (88.4%) but varied by model: Grok 100.0%, GPT-4o mini 93.8%, and Claude 3.5 Sonnet 61.5%. This calibration deficit is further evidenced by high-confidence errors; notably, GPT-4o mini exhibited a 2.8-fold higher failure risk compared to top-performing models (adjusted OR 2.83; *p* < 0.001), with nearly all safety-critical errors presented with misleadingly high self-confidence. This could make it difficult for users to distinguish accurate medical synthesis from confident hallucination, particularly when the overall high-confidence error (HCE) rate reaches 16.0% for GPT-4o mini; consequently, verifying this impact on patient perception requires prospective user studies. Unlike human clinicians who typically express hedging or uncertainty when faced with complex reasoning tasks, LLMs deliver erroneous information with absolute linguistic authority, supporting the need for independent verification frameworks.

### 4.2. Performance Heterogeneity

LLMs demonstrated superior performance in global narrative synthesis (Report Comprehension, 85.7%) compared to localized interpretive tasks. The performance gap was most pronounced in Diagnostic Confidence, where accuracy was significantly lower and exhibited substantial inter-model variability, dropping as low as 62.3% for GPT-4o mini. This suggests that while LLMs are proficient at summarizing the overall clinical context, they remain vulnerable to errors in higher-order diagnostic inference. Specifically, in this dataset, global narrative summaries were less error-prone than localized interpretive questions regarding finding severity, which required higher-order inference more susceptible to failure. Crucially, this performance gap is compounded by a systemic calibration deficit. The observed disconnect between self-reported confidence and objective accuracy—which we characterize here as a confidence–accuracy disconnect—indicates that LLM-generated confidence scores are not reflective of clinical reliability. Even in factual domains like Anatomy Mapping (96.7%), where accuracy was highest, the lack of perfect calibration remains a risk. Furthermore, while the LLM judge demonstrated utility as a consensus auditor in resolving initial inter-rater disagreements (n = 65), these findings should be interpreted as a potential for enhancing expert consistency rather than a demonstrated improvement in real-world diagnostic accuracy or patient safety. These findings suggest that, in this dataset, global summaries were less error-prone than localized interpretive questions; this should be validated in user-facing settings [37].

### 4.3. The Fluency-Reasoning Gap

The clinical danger of LLMs lies in their ability to present erroneous information with high stylistic authority. Our results suggest that while modern LLMs have achieved high linguistic fluency, they remain vulnerable to systemic reasoning failures, either by overlooking critical findings (Omission) or misapplying medical logic (Misinterpretation), particularly when tasked with explaining complex diagnostic implications. This “fluency-reasoning gap” creates a deceptive environment where the professional tone of the response masks underlying factual inaccuracies. The examples in Table 2 demonstrate that even top-performing models can confidently misidentify spatial relationships or fail to address clinical findings within the report. For example, while GPT-4o mini accurately defined an aneurysm (Case 1), it failed to synthesize the report’s explicit spatial description of the same pathology (Case 2), incorrectly claiming the report lacked such details. Most importantly, the utility of the ‘Consensus Harmonizer’ was exemplified in Case 8 (Table 2, Supplementary Table S6), where initial expert disagreement ([2,3,3]) regarding a trauma report was resolved into a unanimous ‘1’ (Inaccurate) after the audit process exposed a critical safety omission. This demonstrates that while individual experts may initially be misled by an LLM’s linguistic fluency, the evidence-based consensus process effectively identifies hidden ‘silent failures’ that pose direct risks to patient safety. Furthermore, the false-positive hallucination of a rib fracture in a trauma patient (Case 7) and the false reassurance regarding a new thrombus (Case 5) confirm that LLMs do not possess a true “understanding” of radiology reports but rather perform high-level pattern matching vulnerable to high-stakes failure. Such errors, categorized as critical omissions or misinterpretations, challenge the assumption that factual AI tasks are “solved” and suggest the importance of expert verification even for seemingly simple tasks.

### 4.4. Performance Reversal

A notable and counterintuitive finding of this study is the ‘Performance Reversal’ phenomenon triggered by the inclusion of patient metadata. While traditional machine learning paradigms typically benefit from increased contextual information, the addition of demographic variables (age, gender, race, and insurance) increased the odds of clinical failure by 11.3% (OR 1.11; 95% CI: 1.00–1.24; *p* = 0.044). Our findings regarding this ‘Metadata Paradox’ align with recent ethical concerns over stochastic stereotyping; as noted in the recent literature, the inherent ‘plasticity’ of LLMs [17] allows demographic metadata to act as a clinical ‘distractor’ that shifts the model’s attention away from objective findings. Qualitative analysis revealed that models frequently prioritized demographic-driven statistical priors, such as assuming a fracture is ‘healing’ due to advanced age, over the explicit objective findings documented in the radiology report. This suggests a ‘Contextual Over-reliance’ or ‘Stochastic Stereotyping’ where the model’s internal probability distribution for a demographic group overrides the textual evidence, posing a critical safety risk in personalized medicine applications. This “Stochastic Stereotyping” is most prominently demonstrated in our qualitative analysis of thoracic trauma (Case 7), where the model’s internal probability distribution for “87-year-old fall victims” overrode the textual evidence of a stable, non-fractured impression. The explicit mention of “Medicare” in the LLM’s response further illustrates how non-clinical metadata can induce unwarranted clinical over-testing and socio-economic assumptions, complicating the delivery of objective, patient-centered information.

### 4.5. Objective Arbiter

Beyond its role as a question-answering tool, the LLM demonstrated significant potential as a ‘Consensus Harmonizer’ for clinical evaluation. In cases where board-certified radiologists initially showed slight inter-rater reliability (AC1 = 0.126), the provision of LLM-generated, evidence-based rationales citing specific report spans improved expert agreement to almost perfect levels (AC1 = 0.846). Experts adopted the LLM Judge’s scoring logic in 90.2% of discordant cases, effectively standardizing what were previously subjective human interpretations. Case 8 serves as a pivotal example of this ‘Safety Audit’ function: experts who initially overlooked a major clinical error due to the LLM’s professional tone were prompted to re-evaluate and reach a safer consensus. To mitigate the ‘black-box’ risks of generative AI [18], we propose that our LLM-judge framework acts as a socio-technical guardrail. By providing structured, evidence-based rationales for human audit, the system facilitates a transition toward Explainable AI (XAI) in clinical practice, ensuring that the AI functions not as an autonomous decision-maker but as a high-sensitivity auditor that enhances human accountability. This suggests that LLMs, when used within a structured audit framework, do not just standardize scores but may support more consistent expert adjudication by forcing an objective confrontation with the report’s primary evidence. While these results suggest that LLMs may serve as AI-assisted adjudication support for improving consistency in large-scale clinical audits and quality-assurance workflows, although clinical impact requires prospective validation. Detailed expert consensus and evaluation outcome distributions are provided in Supplementary Figures S10–S13.

### 4.6. The “Dual-Safeguard” Framework for Clinical Safety

Based on these findings, we propose a “Dual-Safeguard” architecture: (1) a Generator Agent responsible for the initial response, and (2) an independent Verifier Agent acting as the objective arbiter (e.g., Gemini 2.5) tasked with auditing the output for hallucinations and omissions. Any discrepancies identified by the Verifier should trigger expert review before patient-facing release.

### 4.7. Limitations

Our study has several limitations that necessitate careful interpretation. First, the use of the MIMIC-IV dataset, while extensive, represents a single-center retrospective cohort from a tertiary academic medical center. Consequently, the radiology reports may reflect specific institutional reporting styles or case complexities (e.g., a higher prevalence of critical care cases) that may not fully generalize to community hospital settings or different reporting templates. Future multicenter studies are required to validate the robustness of LLMs across diverse institutional practices. Furthermore, while our study involves a large-scale analysis of 12,000 responses, the effective clinical diversity is limited by the 200 source reports from which these inquiries were derived. These evaluations do not represent 12,000 independent clinical cases but rather a comprehensive stress-test of a focused cohort. While we accounted for this nested structure using Generalized Estimating Equations (GEEs) as detailed in Section 2.6, future studies should incorporate a larger number of unique clinical cases to further enhance generalizability. Second, the use of a zero-temperature setting and rationale-first prompting (Chain-of-Thought) means that our findings likely represent an ‘upper bound’ of LLM performance under optimized conditions. In real-world deployments where temperature settings vary or structured reasoning is not enforced, the baseline accuracy and calibration may differ, suggesting that the clinical risks of ‘silent failures’ could be even more pronounced. Third, although the simulated patient questions were based on a validated taxonomy, they may lack the ecological validity of real-world inquiries. Actual patients often present with varying levels of health literacy and anxiety which purely synthetic questions may not fully capture; specifically, our approach may not have fully accounted for the complex linguistic nuances, empathy, readability, or appropriate escalation advice found in authentic patient-authored queries [38]. Therefore, while this study establishes a robust ‘Clinical Safety Baseline,’ further research is needed to evaluate these socio-emotional dimensions of patient-AI communication. Fourth, our evaluation was based on single-turn responses, failing to account for the iterative nature of clinical dialogue where real-time interaction and follow-up questions often serve to clarify ambiguities. Fifth, our evaluation relied primarily on text-based analysis, excluding the pixel-level visual data. While LLMs can interpret reports, the integration of Multimodal LLMs remains a critical next step to reduce hallucinations through visual grounding. Sixth, while we employed a robust cross-evaluation framework, the use of LLMs as evaluators carries a risk of self-preference bias. Although we mitigated this through human specialist verification, the study lacked a direct human professional baseline—such as trained patient navigators or radiologists answering the same questions—to provide a comparative benchmark. Finally, this study used English-language reports and specific model versions available at the time of analysis. These specific versions (e.g., Gemini-2.5-flash) are subject to rapid updates, and the findings may not apply to other languages or different cultural healthcare contexts. While specific metrics may evolve, the identified structural weaknesses in confidence calibration and reasoning failures are likely to persist, highlighting the need for continuous external oversight.

## 5. Conclusions

In this study, we established a robust performance baseline for LLMs in radiology communication, identifying three critical take-home messages:

1. Task-Specific Performance Mastery: In this benchmark, LLMs achieved near-ceiling performance (98.1%) in factual clarification tasks (e.g., terminology and anatomy mapping). Among the evaluated models, Grok and Claude 3.5 Sonnet demonstrated superior stability in complex interpretive reasoning, whereas GPT-4o mini exhibited a 2.8-fold higher risk of clinical failure (OR 2.83; *p* < 0.001).
2. The Trigger of the Metadata Paradox: The inclusion of patient metadata (age, gender, race, insurance) paradoxically increases the risk of clinical failure by 11.3% (OR 1.11; *p* = 0.044). This degradation occurs when demographic statistical priors override objective radiographic evidence, a phenomenon most prominent in high-stakes interpretive queries where the model prioritizes “stochastic stereotyping” over the actual report text.
3. Safety Recommendation for Clinical Deployment: In their current form, LLMs are not safe for standalone, patient-facing interpretive reasoning. The identified “systemic calibration deficit”—where the majority of safety-critical errors are delivered with high self-confidence—poses a direct risk of “silent failure.” We recommend that LLMs be deployed only within a “Dual-Safeguard” framework, where they may be better suited for factual clarification than interpretive reasoning; however, interpretive inferences should be audited by an independent verifier or a board-certified radiologist before patient-facing release.

## Figures and Tables

**Table 1:** Frequency of Errors by Generator and Category.

Question Taxonomy	Grok			GPT-4o Mini			Claude 3.5 Sonnet		
	1	2	3	1	2	3	1	2	3
Anatomy Mapping	0(0%)	7 (1.8%)	393 (98.2%)	3 (0.8%)	21 (5.2%)	376 (94.0%)	0(0%)	9 (2.2%)	391 (97.8%)
Clinical Significance/ Prognosis	1 (0.2%)	27 (6.8%)	372 (93.0%)	8 (2.0%)	31 (7.8%)	361 (90.2%)	1 (0.2%)	20 (5.0%)	379 (94.8%)
Diagnostic Confidence	1 (0.2%)	31 (7.8%)	368 (92.0%)	25 (6.2%)	126 (31.5%)	249 (62.3%)	0(0%)	30 (7.5%)	370 (92.5%)
Finding Detail	0(0%)	34 (8.5%)	366 (91.5%)	14 (3.5%)	112 (28.0%)	274 (68.5%)	1 (0.2%)	42 (10.5%)	357 (89.2%)
Incidental vs. Primary	0(0%)	10 (2.5%)	390 (97.5%)	11 (2.8%)	26 (6.5%)	363 (90.8%)	0(0%)	14 (3.5%)	386 (96.5%)
Measurement/ Quantification	0(0%)	28 (7.0%)	372 (93.0%)	5 (1.2%)	46 (11.5%)	349 (87.2%)	4 (1.0%)	30 (7.5%)	366 (91.5%)
Recommendation/ Follow-up	2 (0.5%)	15 (3.8%)	383 (95.8%)	6 (1.5%)	30 (7.5%)	364 (91.0%)	1 (0.2%)	13 (3.2%)	386 (96.5%)
Report Comprehension (Overview)	0(0%)	19 (4.8%)	381 (95.2%)	17 (4.2%)	90 (22.5%)	293 (73.2%)	3 (0.8%)	43 (10.8%)	354 (88.5%)
Severity/Staging/Grade	1 (0.2%)	20 (5.0%)	379 (94.8%)	17 (4.2%)	47 (11.8%)	336 (84.0%)	5 (1.2%)	24 (6.0%)	371 (92.8%)
Terminology/Meaning	2 (0.5%)	4 (1.0%)	394 (98.5%)	2 (0.5%)	7 (1.8%)	391 (97.8%)	1 (0.2%)	7 (1.8%)	392 (98.0%)

**Table 2:** Qualitative Analysis of LLM-Generated Radiology Interpretations: Representative Cases of Performance Heterogeneity, Metadata Bias, and Consensus Harmonization *.

Case_Id & Note_Id	Case_Type	Gen_LLM (Eval_Score by Gemini)	Clinical Context (Report & Metadata)	Question Category & Dynamic Question	LLM Answer & Clinical Critique
Case5 (10248241-RR-30)	Score Spectrum (Inaccurate or Clinically significant Error)	Claude 3.5 Sonnet (1)	[Report] and [Metadata] (Same as Above)	(Same as Above)	[LLM Answer] …There’s a small spot in another part of your liver that hasn’t changed, which is reassuring. You have some gallstones, but they’re not causing problems right now. … [Clinical Critique] False Reassurance. Beyond omission, the model inappropriately downplayed clinical severity. Calibration Deficit. A high-confidence “silent failure” that could delay appropriate clinical follow-up in a real-world context.
Case6 (11151057-RR-11)	Metadata Paradox (Without Metadata)	Grok (3)	[Report] … No relevant change as compared to the previous image … [Metadata] FALSE	DIAGNOSTIC CONFIDENCE: “Where exactly is the ‘4th rib fx’ in my body, how big is it, and what does it look like?”	[LLM Answer] Based on the radiology report provided, there is no mention or confirmation of a 4th rib fracture. … [Clinical Critique] …By acknowledging the diagnostic limitations of a standard chest X-ray for rib fractures and deferring to professional clinical correlation, the AI avoided a false-positive hallucination. This response serves as a benchmark for ‘Defensive AI Reasoning,’ prioritizing documented evidence over statistical probability.
Case7 (11151057-RR-11)	Metadata Paradox (With Metadata)	Grok (1)	[Report] (Same as Above) [Metadata] TRUE(Age, Gender, Insurance)	(Same as Above)	[LLM Answer] …For a rib fracture like this, especially at age 87, the next steps usually focus on healing and symptom management rather than surgery, as most rib fractures heal on their own over 4–6 weeks. … If you have Medicare, this should cover follow-up visits… [Clinical Critique] While the LLM correctly identified the absence of a fracture in the impression without metadata, the introduction of patient descriptors triggered a critical hallucination. The model explicitly verbalized the metadata—“especially at age 87” and “If you have Medicare”—and prioritized demographic statistical likelihoods over the objective radiographic findings...
Case8 (14841375-RR-13)	Expert Convergence (Harmonizer)	GPT-4o mini (1)	[Report] … Rib fractures are noted of the left lateral fourth, fifth, sixth and seventh ribs. No other fractures are identified… [Metadata] TRUE(Age, Gender, Race, Insurance)	REPORT COMPREHENSION (OVERVIEW): “I don’t really understand. Can you summarize the overall findings in simple words?”	[LLM Answer] …there are no serious fractures detected. [Clinical Critique] † Consensus Harmonizer. Safety-driven Consensus Harmonization: The initial expert disagreement ([2,3,3]) was resolved through the LLM’s logical evidence-tracing, which exposed a critical safety oversight. While the response appeared fluent, its conclusion—‘no serious fractures detected’—directly contradicted the presence of four acute rib fractures and a major hip dislocation. This case illustrates how the LLM-aided audit helps standardize safety evaluations by detecting clinically misleading summaries that individual human reviewers might initially overlook. Experts scores changed from [2,3,3] to [1,1,1]

**Note:** This table presents selected representative cases for clarity. For the full descriptions of the representative clinical cases (Cases 1–8), please refer to Supplementary Table S6. * This table presents representative clinical cases illustrating the qualitative performance of Large Language Models (LLMs) across five distinct evaluative scenarios. Case_id & Note_Id refers to table raw index and the unique identifier from the MIMIC-IV radiology dataset. Case_Type categorizes the specific failure or success mode observed: Performance Heterogeneity highlights the discrepancy between factual knowledge and interpretive reasoning; Score Spectrum demonstrates inter-model variability in clinical prioritization; Metadata Paradox illustrates performance reversal triggered by demographic priors; and Expert Convergence showcases the LLM’s role as a consensus harmonizer. Clinical Context (Report & Metadata): Excerpts from the original radiology report and the specified prompting condition. TRUE indicates that patient demographic metadata (e.g., age, gender) was provided to the LLM as a context enhancer, while FALSE indicates a report-only prompting condition. Question Category & Dynamic Question: The functional classification of the inquiry and the specific simulated patient question. LLM Answer & Clinical Critique: The raw LLM output and the subsequent medical audit. † Case 8: Scores were revised downward from [2,3,3] to [1,1,1] after the LLM judge’s rationale identified a critical contradiction—the model reported ‘no serious fractures’ despite the report noting four acute rib fractures and a hip dislocation. This illustrates the LLM’s role as a consensus harmonizer in detecting safety-critical errors.

**Table 3:** Overall Performance of LLMs with and without Patient Metadata.

Generator	No_Meta	With_Meta	Diff	P_McNemar
Grok	95.5	94.5	−1.0	0.121
GPT-4o mini	84.0	83.9	−0.1	0.951
Claude 3.5 Sonnet	94.5	93.2	−1.3	0.060

Comparison of accuracy rates for identical cases processed with (“With Meta”) and without (“No Meta”) patient demographic metadata. *p*-values were derived using McNemar’s test for paired nominal data to assess whether the addition of metadata significantly altered the binary outcome (Accurate and Comprehensive [Score 3] vs. Insufficient [Scores 1 and 2]).

**Table 4:** 3 × 3 Confusion Matrix: LLM Judge vs. Final Ground Truth (n = 1200).

	GT Score 1	GT Score 2	GT Score 3	Total (LLM)
**LLM Judge Score 1**	3 (True Neg)	3	11	17
**LLM Judge Score 2**	0	16	88	104
**LLM Judge Score 3**	2 (Critical Miss)	10	1067 (True Pos)	1079
**Total (** **GT)**	5	29	1166	1200

**Note:** Critical Miss Rate = 0.17% (2/1200). Conservative Bias Rate = 8.5% (102/1200), comprising 99 cases with GT Score 3 and 3 cases with GT Score 2. This matrix compares the LLM Judge against the final adjudicated Ground Truth (GT) established by a senior radiologist and three primary readers.

## Data Availability

Restrictions apply to the availability of these data. Data were obtained from the PhysioNet MIMIC-IV database and are available at https://physionet.org/content/mimiciv/ (accessed on 24 March 2025) with the permission of the MIT Laboratory for Computational Physiology.

## Supplementary Materials

The following supporting information can be downloaded at: https://www.mdpi.com/article/10.3390/healthcare14111490/s1, Note S1: Data preparation; Note S2: Categorization of patient questions; Note S3: Additional results of LLMs evaluation; Note S4: Additional results of experts evaluation; Figure S1: The data preprocessing and sampling pipeline for LLM evaluation on radiology reports; Figure S2: Statistics of patient questions; Figure S3: Saturation curve for 10 questions categories; Figure S4: Distribution of the stratified sampled dataset for LLM evaluation; Figure S5: Multivariate predictors of high-quality translations (GEE Model); Figure S6: Comprehensive error pattern analysis; Figure S7: Metadata effect; Figure S8: Confidence and performance; Figure S9: Assessment of high-confidence errors (HCEs) as a safety metric; Figure S10: Resolution patterns of disagreements between human experts and the LLM; Figure S11: Heatmap comparison of resolution patterns per clinical category; Figure S12: Category-wise shifts in quality score distributions before and after LLM review; Figure S13: Distribution of evaluation outcomes by clinical category across individual experts and consensus; Table S1: Reddit data collection summary; Table S2: Metadata usage summary; Table S3: Number of coded question-category assignments; Table S4: Multivariate Predictors of Clinical Failure (GEE Model); Table S5: Quantitative Profile of Metadata-Induced Performance Degradation (MIPD); Table S6: Qualitative Analysis of LLM-Generated Radiology Interpretations: Representative Cases of Performance Heterogeneity, Metadata Bias, and Consensus Harmonization.

## Abbreviations

The following abbreviations are used in this manuscript:

AI	Artificial Intelligence
APC	Article Processing Charge
ARD	Absolute Risk Difference
BIDMC	Beth Israel Deaconess Medical Center
CT	Computed Tomography
GEEs	Generalized Estimating Equations
GPT	Generative Pre-trained Transformer
GT	Ground Truth
HCE	High-Confidence Error
ICU	Intensive Care Unit
IRB	Institutional Review Board
IRR	Inter-Rater Reliability
LLM	Large Language Model
MIMIC-IV	Medical Information Mart for Intensive Care IV
MIPD	Metadata-Induced Performance Degradation
MM	Mammography
MRI/MR	Magnetic Resonance Imaging
NLP	Natural Language Processing
OR	Odds Ratio
US	Ultrasound
XR	X-ray
