# Supplementary material for: Patient-Facing Radiology Communication with LLMs: Calibration Deficit and the Metadata Paradox
Source: Healthcare (Basel). 2026 May 27;14(11):1490. doi: 10.3390/healthcare14111490 (PMC13257061; doi:10.3390/healthcare14111490)
Supplement: Supplementary file 1 [file healthcare-14-01490-s001.zip › healthcare-4229479-supplementary-format-corrected pages.pdf]

Supplementary Materials

Patient-Facing Radiology Communication with LLMs: Calibration Deficit and the Metadata Paradox

Cheong Shin [1], Jung Hyun Park [1,2,3,*], Sungjun Kim [1,3,4,5], Young Han Lee [5,6] and Hong-Seon Lee [4,*]

[1] Department of Integrative Medicine, The Graduate School, College of Medicine, Yonsei University, Seoul 03722, Republic of Korea; royalblue@yonsei.ac.kr (C.S.); agn70@yuhs.ac (S.K.)
[2] Department of Rehabilitation Medicine, Gangnam Severance Hospital, Rehabilitation Institute of Neuromuscular Disease, College of Medicine, Yonsei University, 211, Eonju-ro, Gangnam-gu, Seoul 03722, Republic of Korea
[3] Department of Medical Device Engineering and Management, The Graduate School, College of Medicine, Yonsei University, Seoul 03722, Republic of Korea
[4] Department of Radiology, Gangnam Severance Hospital, College of Medicine, Yonsei University, Seoul 03722, Republic of Korea
[5] Institute for Innovation in Digital Healthcare, Yonsei University, Seoul 03722, Republic of Korea; sando@yuhs.ac
[6] Department of Radiology, Research Institute of Radiological Science and Center for Clinical Imaging Data Science, Severance Hospital, College of Medicine, Yonsei University, Seoul 03722, Republic of Korea
* Correspondence: rmpjh@yuhs.ac (J.H.P.); reanhea55@yuhs.ac (H.-S.L.)

Contents

**Note S1. Data preparation**	**............**	**1**
Figure S1. The data preprocessing and sampling pipeline for LLM evaluation on radiology reports.	....	1
**Note S2. Categorization of Patient Questions**	**............**	**3**
Table S1. Reddit Data Collection Summary	....	4
Table S2. Metadata usage summary	....	4
Table S3. Number of coded question-category assignments	....	5
Figure S2. Statistics of Patients Questions	....	6
Figure S3. Saturation Curve for 10 Questions categories	....	7
**Note S3. Additional Results of LLMs Evaluation**	**............**	**9**
Figure S4. Distribution of the Stratified Sampled Dataset for LLM Evaluation.	....	9
Figure S5. Multivariate Predictors of Clinical Failure (GEE Model).	....	11
Figure S6. Comprehensive Error Pattern Analysis	....	12
Figure S7. Metadata Effect	....	13
Figure S8. Confidence and Performance	....	14
Figure S9. Assessment of High-Confidence Errors (HCEs) as a safety metric	....	15
Table S4. Multivariate Predictors of Clinical Failure (GEE Model).	....	16
Table S5. Quantitative Profile of Metadata-Induced Performance Degradation (MIPD)	....	18
**Note S4. Additional Results of Experts Evaluation**	**............**	**19**
Figure S10. Resolution patterns of disagreements between human experts and the LLM	....	19
Figure S11. Heatmap comparison of resolution patterns per clinical category	....	20
Figure S12. Category-wise shifts in quality score distributions before and after LLM review	....	21
Figure S13. Distribution of evaluation outcomes by clinical category across individual experts and consensus	....	23
Table S6. Qualitative Analysis of LLM-Generated Radiology Interpretations: Representative Cases of Performance Heterogeneity, Metadata Bias, and Consensus Harmonization.	....	24

Note S1. Data preparation

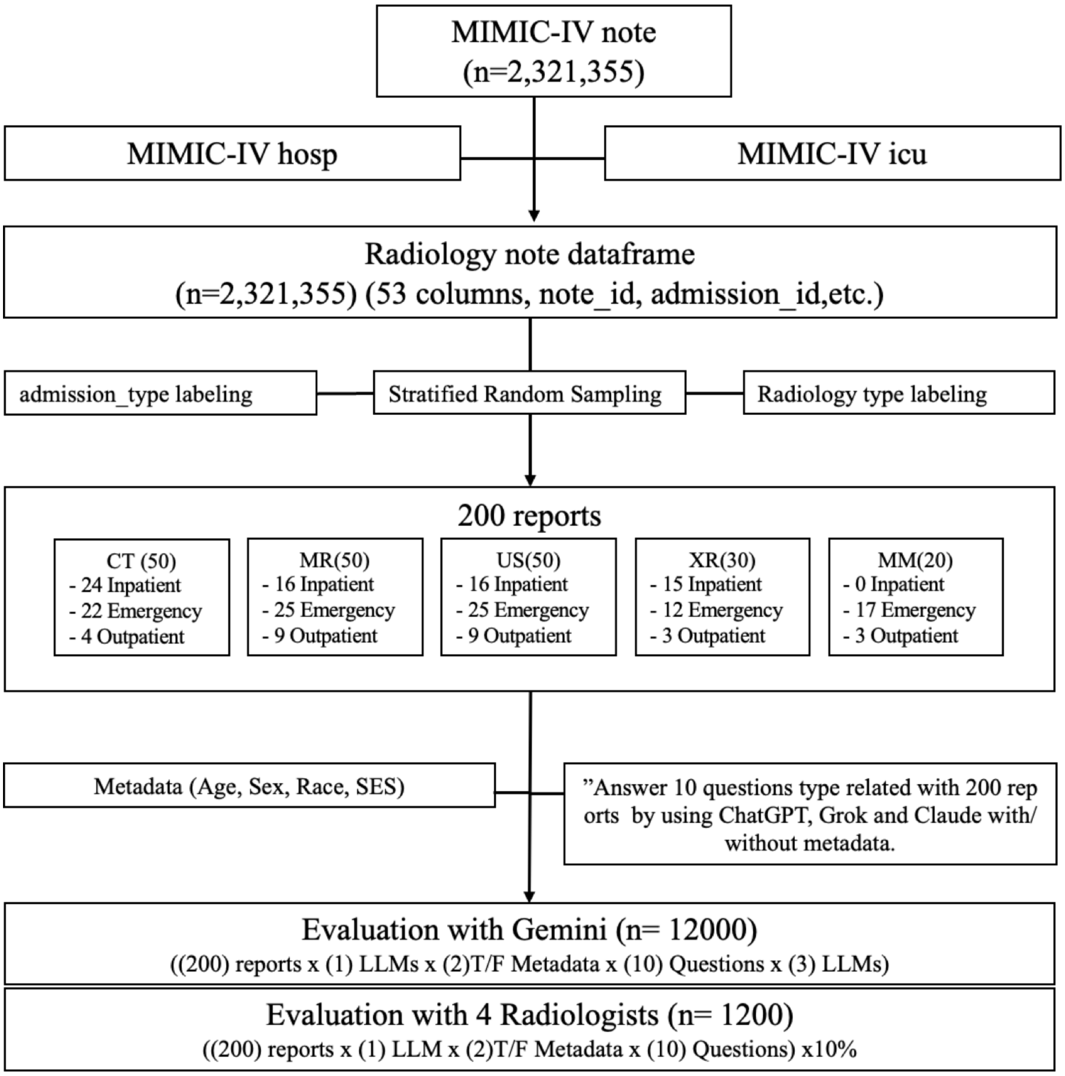

Figure S1. The data preprocessing and sampling pipeline for LLM evaluation on radiology reports.

This flowchart illustrates the multi-stage methodology used to create a meticulously curated dataset for evaluating Large Language Models (LLMs) in the

context of radiology report analysis. MIMIC-IV Note: The process begins with the raw MIMIC-IV clinical notes, comprising over 2.3 million records from both hospital and ICU settings.

Radiology Note Dataframe: A specialized dataframe containing 53 key columns (including note_id, admission_type, and others) was extracted from the initial dataset.

Data Labeling and Stratified Sampling: This critical phase involved two parallel processes:

Admission Type Labeling: Each note was classified into one of three distinct admission types: Inpatient, Outpatient, or Emergency.

Radiology Type Labeling: Reports were meticulously labeled and grouped into five primary radiology subgroups: CT, MR, US, XR, and MM. Targeted Stratified Random Sampling: To ensure a balanced representation, a stratified random sampling method was applied, resulting in a total of 200 reports. This approach ensured that each radiology subgroup met predefined modality quotas, providing sufficient representation for each category rather than following simple proportional distribution.

Final Sampled Dataset (200 reports): The final dataset of 200 reports was constructed with a specific target count for each modality (CT: 50, MR: 50, US: 50, XR: 30, MM: 20), and within each modality, the original distribution of admission types was preserved.

LLM Evaluation: The curated dataset was used to generate 10 questions per report, which were then answered by three different LLMs (GPT-4o mini, Claude 3.5 Sonnet, and Grok) based on 1-3 scale (Score 1 = Inaccurate or Clinically Significant Error; Score 2 = Ambiguous or Incomplete; Score 3 = Accurate and Comprehensive). This process generated a total of 12,000 answers and 12,000 evaluation data points, providing a comprehensive basis for a robust comparative analysis of LLM performance with Gemini. Radiologists Evaluation**:** A 10% subset was independently evaluated by three board-certified radiologists and adjudicated by a four-member expert panel.

Note S2. Categorization of Patient Questions

Data Source and Collection

To investigate the types of questions that laypersons pose regarding radiology reports, we collected publicly available posts from the subreddit *r/AskDocs* using the Reddit API. Posts were retrieved using the keyword search "radiology report" to ensure relevance to imaging findings and patient interpretation. A total of 200 posts were collected. Posts were included if they described a personal radiology report and contained sufficient clinical context to allow classification, and excluded if they were off-topic, advertisements, or contained insufficient information. From these 200 unique posts, a total of 837 distinct question-category assignments were identified, reflecting the multi-label nature of patient inquiries where a single post often contained multiple specific questions.

Data Preprocessing and Ethics

All personally identifiable information was removed to protect user privacy. Posts were anonymized by removing usernames and other identifying metadata. For each post, we extracted basic metadata such as patient-reported age, gender, race, and insurance type when available. The posts were then structured for further analysis, including classification into question types.

Sample Posts

Representative examples of the collected questions include:

- *Case 1:* "An adult male sought clarification regarding follow-up imaging (MRI and CT) conducted to investigate a suspected bone-related ear condition. While the formal report ruled out the primary concern, it noted anatomical variations involving the semicircular canals and the skull base. Despite having some medical background, the patient found the findings difficult to interpret, as did their primary care physician."
- *Case 2:* "A patient with a significant history of gynecological surgeries reported recurring pelvic pain following a recent procedure. A follow-up ultrasound identified a mid-sized, mildly complex cyst in the adnexal region. The report detailed findings such as internal septations and vascular flow within the cyst wall but noted the absence of solid components or irregular projections."

Rationale for Use

These data were used to:

1. Categorize patient questions according to content type (e.g., interpretation, management advice, prognosis).
2. Identify common patterns and metadata usage (age, gender, race, insurance) to inform subsequent LLM prompt design for patient-centered radiology report translation.

Table S1. Reddit Data Collection Summary.

Total posts	200
Source	r/AskDocs
Search keyword	"radiology report"
Posts included	Personal radiology report posts with clinical context
Posts excluded	Off-topic, advertisements, insufficient information
Representative cases	• "An adult male sought clarification regarding follow-up imaging (MRI and CT) conducted to investigate a suspected bone-related ear condition. While the formal report ruled out the primary concern, it noted anatomical variations involving the semicircular canals and the skull base. Despite having some medical background, the patient found the findings difficult to interpret, as did their primary care physician."

Table S2. Metadata usage summary.

Total Cases	200
Age	179
Gender	180
Race	33
Insurance	8

Table S3. Number of coded question-category assignments.

Qtype	Number of coded assignments
Clinical Significance/Prognosis	244
Recommendation/Follow-up	174
Report Comprehension (Overview)	135
Terminology/Meaning	82
Finding Detail	67
Severity/Staging/Grade	60
Diagnostic Confidence	45
Measurement/Quantification	14
Anatomy Mapping	10
Incidental vs. Primary	6

†Note: The total number of assignments (n=837) exceeds the number of original posts (n=200) because many patient inquiries contained multiple questions that were coded into more than one category (multi-label coding).

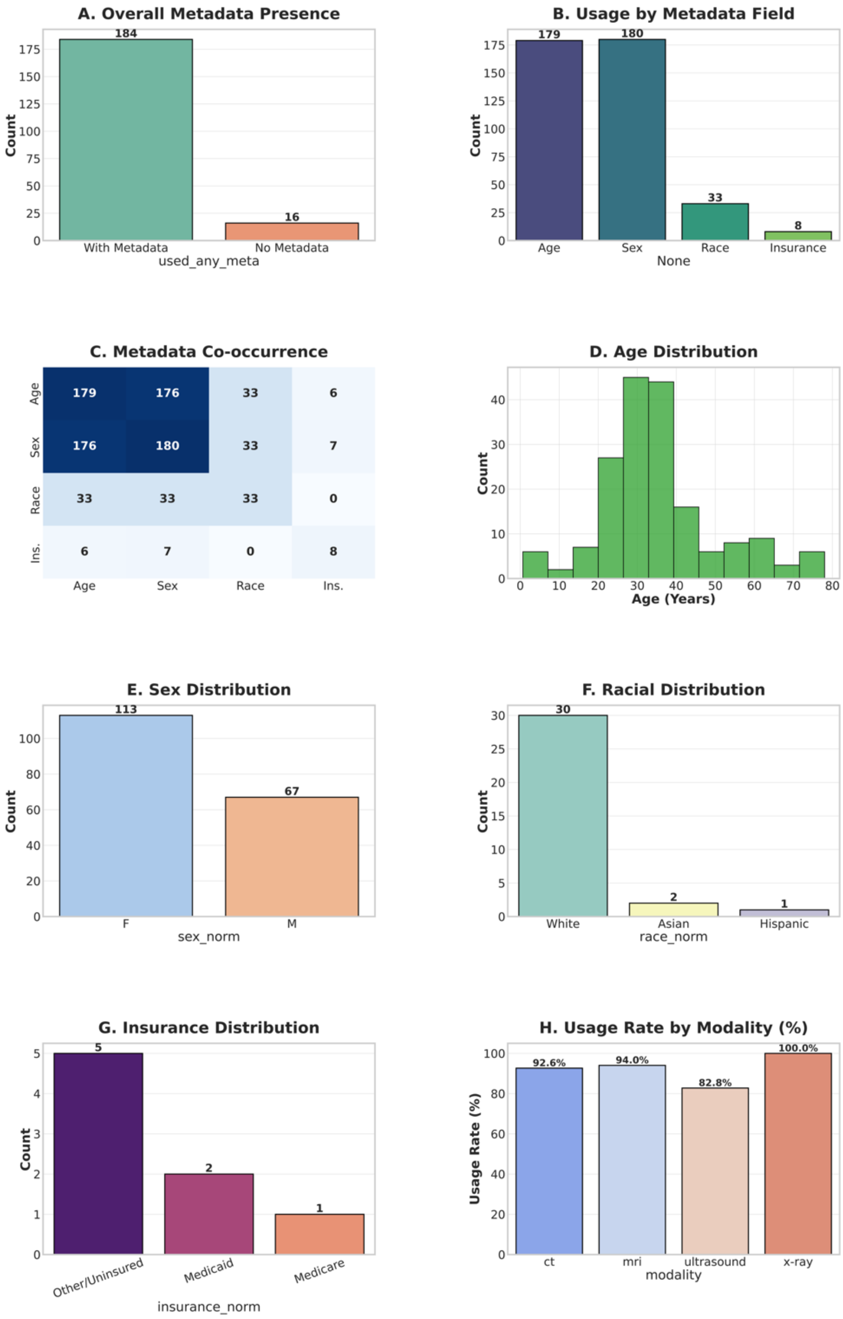

Figure S2. Statistics of Patient Questions.

We performed a cross-validation between the manually annotated metadata flag (has_meta) and the derived metadata presence (used_any_meta). This process identified 10 inconsistencies out of 200 cases, which were subsequently corrected. Through this step, we ensured data quality by detecting and resolving tagging errors prior to analysis.

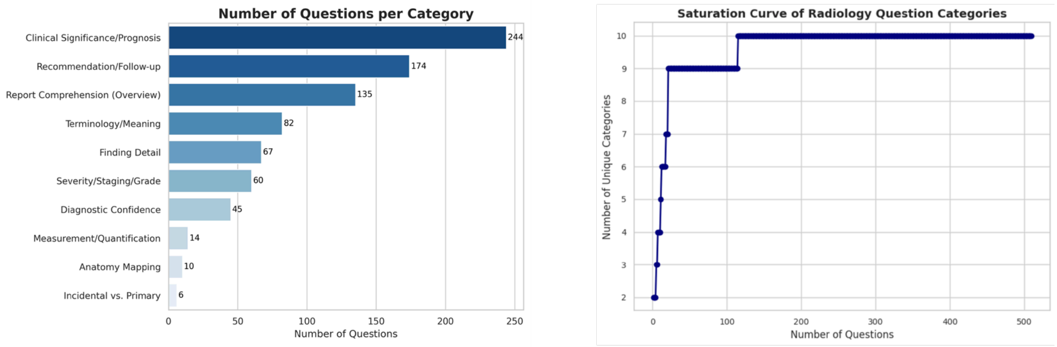

Figure S3. Saturation Curve for 10 Questions categories.

Analysis of Reddit posts regarding radiology reports (N=200 unique posts) yielded a total of 837 coded question-category assignments (assignments per post ranged from 1 to 10). The plotted saturation curve illustrates that thematic saturation was definitively achieved at ten unique categories (C=10).

The most frequently encountered category was Clinical Significance/Prognosis (n=244 assignments; 29.1%), followed by Recommendation/Follow-up (n=174; 20.8%), and Report Comprehension (Overview) (n=135; 16.1%). Other categories included Terminology/Meaning (n=82; 9.8%) and Finding Detail (n=67; 8.0%).

Sparse categories included Measurement/Quantification (n=14; 1.7%), Anatomy Mapping (n=10; 1.2%), and Incidental vs. Primary (n=6; 0.7%). Notably, the top four categories accounted for 75.9% of all coded category assignments (635/837), confirming that these core frameworks encompass the majority of patient concerns within real-world digital discourse. These insights underscore the necessity for enhanced communication strategies in radiology that address the specific interpretive and prognostic informational needs of patients. Percentages for each category are calculated based on the total number of coded question-category assignments (n = 837) rather than the number of unique posts (n = 200). This accounting method reflects the multi-label nature of the data, where a single post

may encompass multiple inquiry types, and ensures that the cumulative percentage across all ten categories equals 100%.

Note S3. Additional Results of LLMs Evaluation

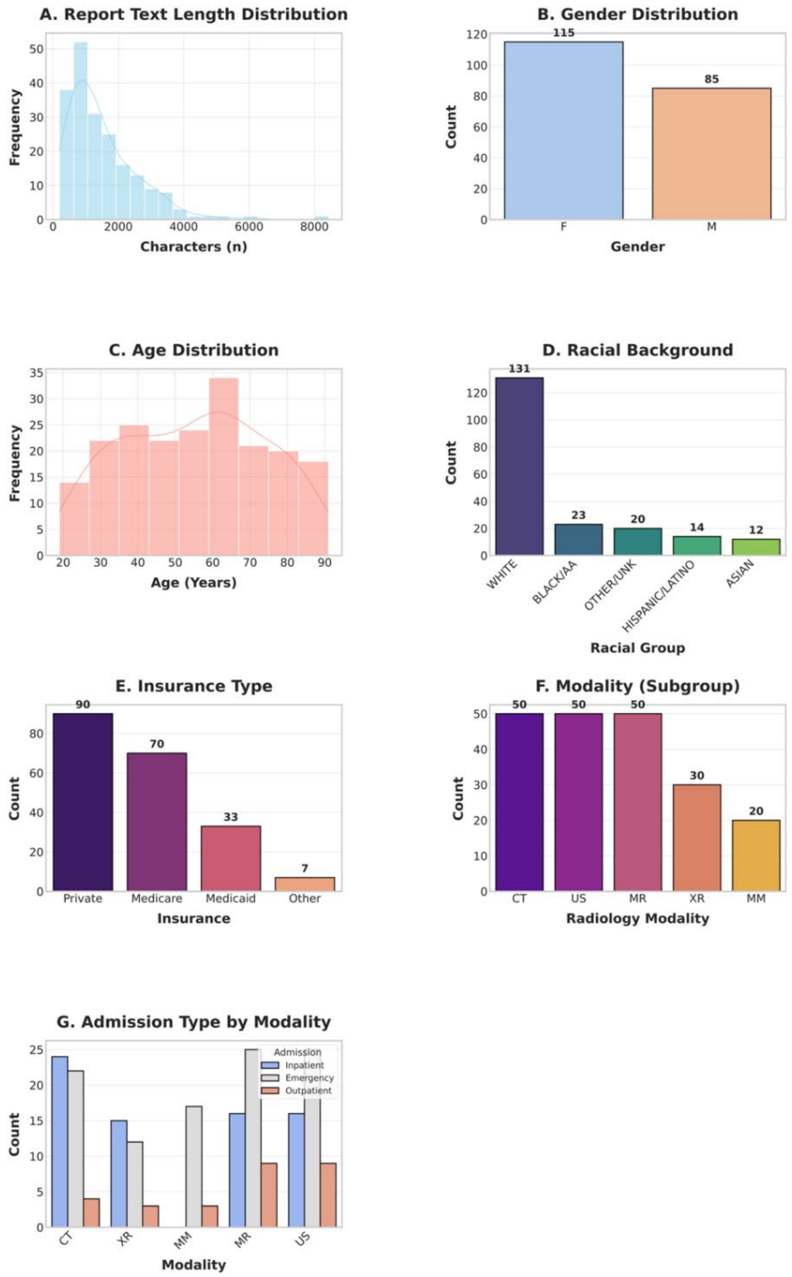

Figure S4. Distribution of the Stratified Sampled Dataset for LLM Evaluation.

The figure presents the key characteristics of the sampled dataset used for evaluating Large Language Models (LLMs) in transforming radiology reports. The data was collected from a clinical repository and meticulously stratified to ensure a representative sample across various patient and report attributes. (A) Distribution of Report Text Length: A histogram illustrating the frequency of radiology reports based on their character count. This provides a quantitative measure of the textual complexity within the dataset, highlighting the variation in report verbosity. (B) Distribution by Gender: A bar chart showing the count of patients by gender. This panel confirms that the dataset includes both male and female patients, which is important for evaluating potential gender-related differences in report content and LLM transformation. (C) Distribution by Age: A histogram depicting the age distribution of the patients in the sample. It provides insight into the age profile of the patient cohort, which can be a critical metadata point in medical report interpretation. (D) Distribution by Race: A bar chart showing the distribution of patients across different race categories. To enhance clarity and standardize the data, various sub-classifications (e.g., 'ASIAN - CHINESE', 'BLACK/CAPE VERDEAN') were grouped into broader categories (e.g., 'ASIAN', 'BLACK/AFRICAN AMERICAN', 'HISPANIC/LATINO'). The "OTHER / UNKNOWN" category aggregates less common groups and unrecorded data. (E) Distribution by Insurance Type: A bar chart displaying the count of patients by insurance type. This shows the demographic diversity of the dataset in terms of patient socioeconomic background. (F) Distribution by Radiology Subgroup: A bar chart representing the number of reports for each radiology modality (CT, XR, MM, MRI, and US). This panel confirms the successful execution of stratified sampling, ensuring a balanced representation of each imaging modality as intended by the study design. G) Distribution of Admission Type by Modality: A grouped bar chart illustrating the count of each classified_admission_type within each radiology subgroup. This panel serves as a crucial validation of the stratification process, demonstrating that the sampling method successfully captured the intended distribution of patient admission types across the different imaging modalities.

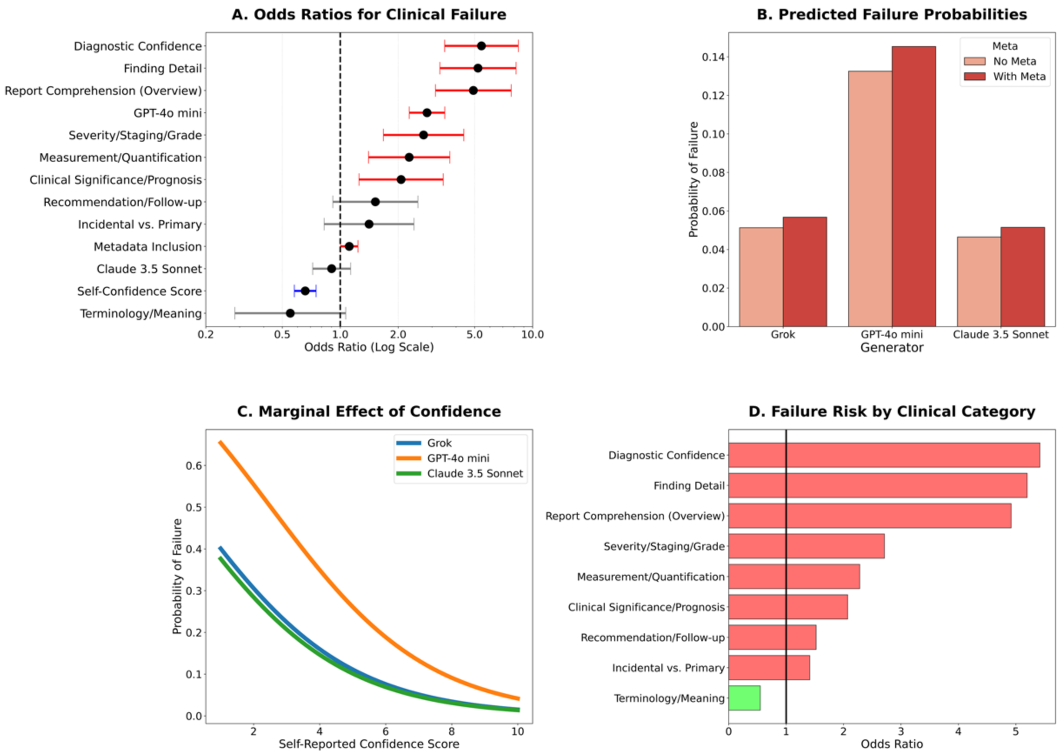

Figure S5. Multivariate Predictors of Clinical Failure (GEE Model). .

(A) Forest plot displaying Odds Ratios (OR) and 95% Confidence Intervals (CI) from the GEE model predicting clinical failure (Score 1 or 2). GPT-4o mini was significantly associated with higher odds of failure compared to the reference (Grok) (OR = 2.83, 95% CI: 2.28–3.49, $p < 0.001$). **(B)** Predicted probabilities of failure for each generator. GPT-4o mini shows a substantially higher risk of providing insufficient responses compared to Grok and Claude 3.5 Sonnet. **(C)** Marginal effect plot showing the predicted probability of failure as self-reported confidence increases. The risk of error drops significantly as the model's internal confidence rises. **(D)** Odds ratios for specific question categories relative to 'Anatomy Mapping'. Categories with OR > 1 indicate a significantly higher risk of failure (e.g., 'Diagnostic Confidence', OR = 5.42, $p < 0.001$).

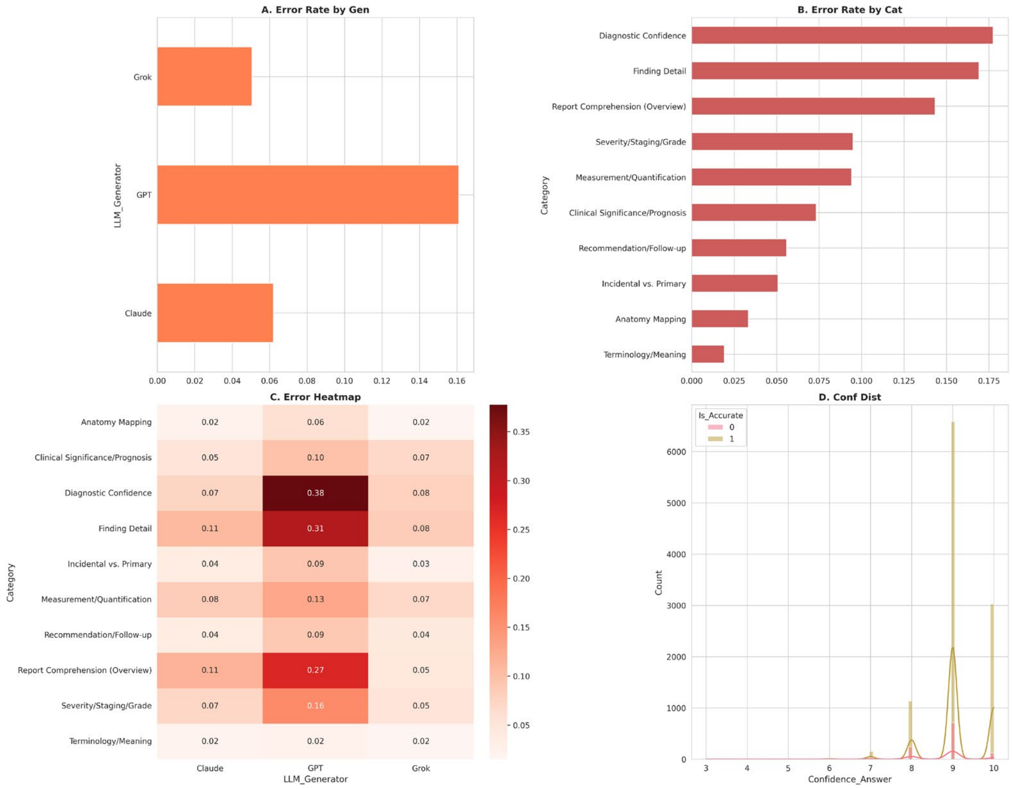

Figure S6. Comprehensive Error Pattern Analysis.

(A) Horizontal bar chart ranking LLMs by total error rate (1 - Accuracy). **(B)** Horizontal bar chart ranking question categories by error rate, identifying 'Diagnostic Confidence' and 'Finding Detail' as the most error-prone domains. **(C)** Detailed heatmap of error rates categorized by Generator and Question Category, providing a granular view of failure modes (e.g., GPT-4o mini's high error rate in 'Report Comprehension'). **(D)** Histogram with kernel density overlay showing the distribution of confidence scores for successful (Accurate) versus failed (Error) translations, reinforcing the finding of high-confidence hallucinations.

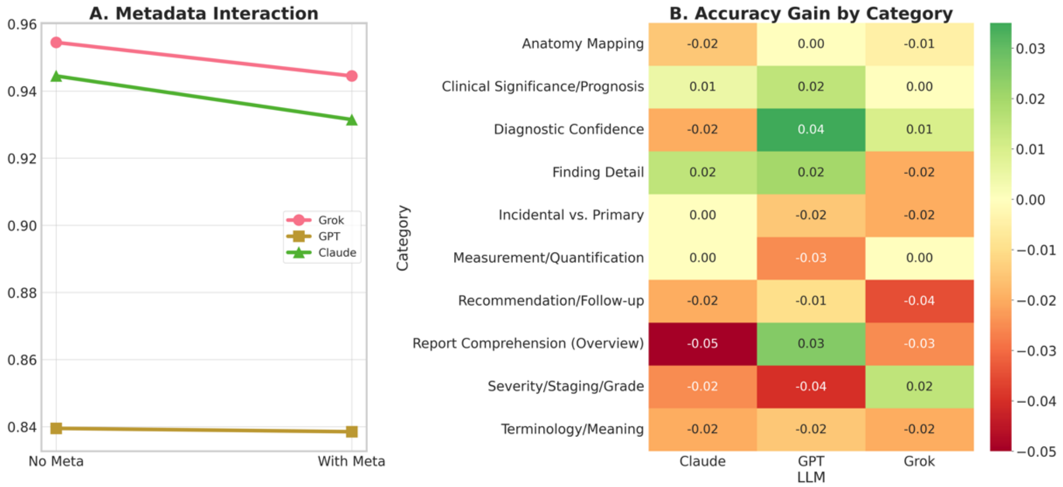

Figure S7. Metadata Effect .

(A) Interaction plot comparing accuracy rates between conditions with and without patient metadata (age, reported gender, race, and insurance) across the three LLMs. Error bars represent 95% bootstrap confidence intervals. Surprisingly, the inclusion of metadata did not lead to statistically significant improvements in accuracy for any model, as determined by McNemar's test for paired nominal data (**Grok:** 95.4% vs. 94.4%, $p = 0.12$; **GPT-4o mini:** 83.95% vs. 83.8%, $p = 0.95$; **Claude 3.5 Sonnet:** 94.4% vs. 93.1%, $p = 0.06$). **(B)** Heatmap illustrating the net gain or loss in accuracy (Accuracy(*with_meta)-Accuracy*(no_meta)) for each question category. Red cells indicate performance degradation, while green cells indicate improvement when metadata was provided.

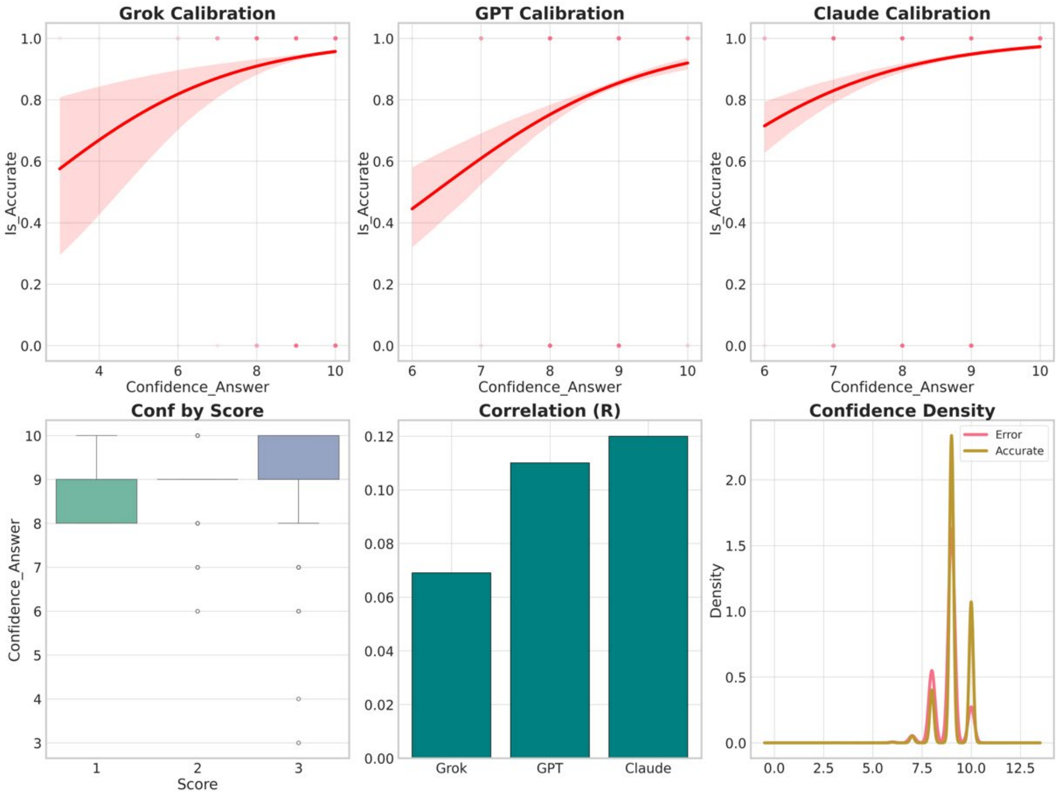

Figure S8. Confidence and Performance.

Analysis of LLM Confidence Calibration. (A–C) Logistic regression curves with scatter plots (jittered) for each model, visualizing the probability of a correct response (Score 3) as a function of the self-reported confidence score (1–10). **(D)** Box plots showing the distribution of confidence scores stratified by evaluation scores (1, 2, 3), demonstrating that confidence generally increases with response quality. **(E)** Bar chart of Pearson correlation coefficients (r) between confidence and accuracy. While positive correlations exist, they remain weak (Claude 3.5 Sonnet: r=0.12; GPT-4o mini: r=0.11; Grok: r=0.07), suggesting suboptimal calibration. **(F)** Kernel density estimation (KDE) plots comparing confidence distributions for Accurate (Score 3) versus Inaccurate (Score 1 & 2) responses. Although accurate responses had significantly higher mean confidence (t-test, $p < 0.001$), considerable overlap indicates frequent high-confidence errors.

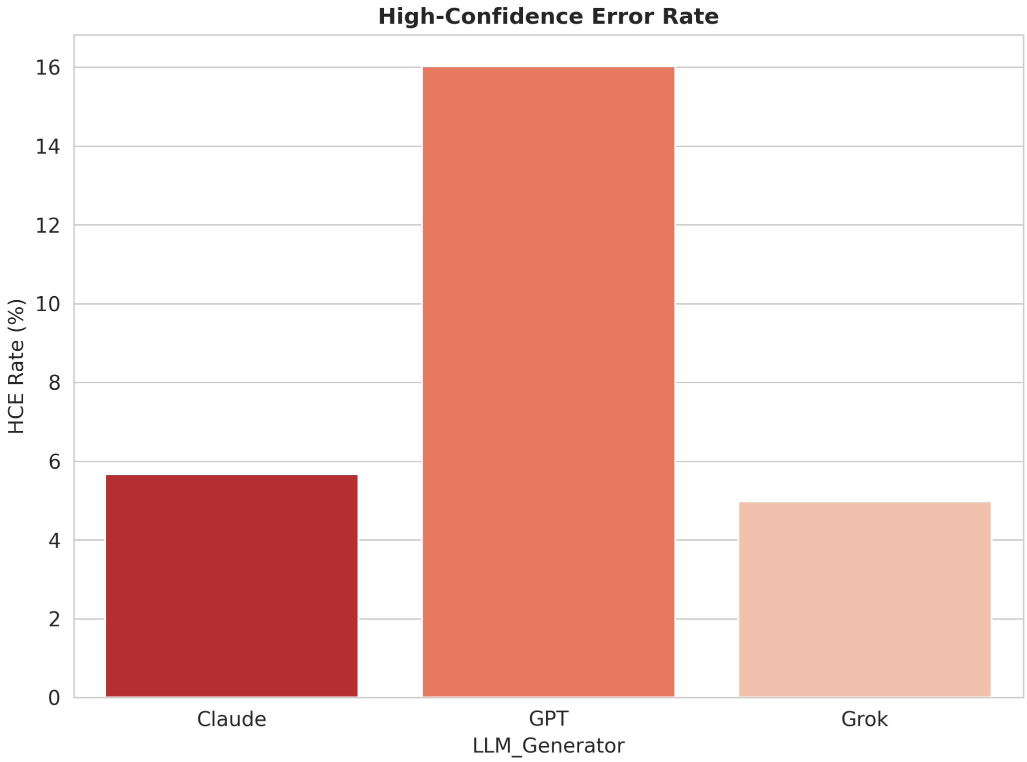

Figure S9. Assessment of High-Confidence Errors (HCE) as a safety metric.

HCE rates represent the frequency of responses where the model generated an erroneous answer (Score 1 or 2) but assigned itself a high confidence score ($\geq 8/10$). GPT-4o mini exhibited a markedly higher HCE rate (16.0%) compared to Claude 3.5 Sonnet (5.7%) and Grok (5.0%) ($p < 0.001$, Pearson χ^2test). This independent evaluation highlights that internal confidence scores are unreliable proxies for safety, particularly for GPT-4o mini, necessitating external validation mechanisms.

Table S4. Multivariate Predictors of Clinical Failure (GEE Model).

index	Coef	SE	CI_L	CI_H	P	OR	OR_L	OR_H
Intercept	0.018	0.681	-1.317	1.354	0.979	1.018	0.268	3.871
C(LLM_Generator)[T.Claude 3.5 Sonnet]	-0.103	0.116	-0.330	0.124	0.374	0.902	0.719	1.132
C(LLM_Generator)[T.GPT-4o mini]	1.039	0.109	0.826	1.251	0.000	2.825	2.284	3.495
C(Use_Meta_Bool)[T.True]	0.107	0.053	0.003	0.211	0.044	1.113	1.003	1.235
C(Category)[T.Clinical Significance/Prognosis]	0.728	0.257	0.224	1.233	0.005	2.071	1.251	3.431
C(Category)[T.Diagnostic Confidence]	1.690	0.225	1.249	2.131	0.000	5.420	3.488	8.422
C(Category)[T.Finding Detail]	1.648	0.233	1.193	2.104	0.000	5.198	3.296	8.199
C(Category)[T.Incidental vs. Primary]	0.345	0.274	-0.193	0.883	0.209	1.412	0.824	2.417
C(Category)[T.Measurement/Quantification]	0.825	0.248	0.339	1.311	0.001	2.282	1.403	3.711
C(Category)[T.Recommendation/Follow-up]	0.420	0.260	-0.089	0.930	0.106	1.522	0.915	2.533
C(Category)[T.Report Comprehension (Overview)]	1.593	0.231	1.140	2.046	0.000	4.918	3.127	7.734
C(Category)[T.Severity/Staging/Grade]	0.997	0.245	0.517	1.478	0.000	2.711	1.676	4.385
C(Category)[T.Terminology/Meaning]	-0.598	0.339	-1.262	0.066	0.077	0.550	0.283	1.068
Confidence_Answer	-0.419	0.066	-0.549	-0.289	0.000	0.657	0.577	0.749

- **Model Specification:** The dependent variable is **binary failure (1 = Insufficient [Score 1 and 2]; 0 = Accurate [Score 3])**. The model adjusts for LLM generator type, patient metadata usage, clinical question categories, and self-reported confidence scores.
- **Reference Groups:** LLM Generator: **Grok**; Metadata: Absent; Category: Anatomy Mapping.
- **Key Interpretations:**
  - **LLM Generators:** GPT-4o mini was a significant **positive predictor of failure** (OR, 2.83; 95% CI: 2.28, 3.49; $p < 0.001$), while Claude 3.5 Sonnet showed no significant difference from the reference Grok ($p = 0.374$).
  - **Metadata Usage:** Inclusion of patient metadata was associated with a **11.3% increase in the odds of failure** (OR, 1.11; 95% CI: 1.00, 1.24; $p = 0.044$).
  - **Clinical Domain:** Interpretive tasks were significantly more prone to errors; 'Diagnostic Confidence' questions showed the **highest risk of failure (OR, 5.42; $p < 0.001$)** relative to Anatomy Mapping.
  - **Confidence Calibration:** Higher self-reported confidence was a **strong negative predictor of failure (OR, 0.66 per unit increase; $p < 0.001$)**, indicating that errors are more likely to occur when the model identifies low certainty.

Note—CI = confidence interval, Coef = regression coefficient, OR = odds ratio, SE = standard error.

Table S5. Quantitative Profile of Metadata-Induced Performance Degradation (MIPD).

LLM Generator	MIPD Count (n)	Hallucination in MIPD (%)	Primary Distractor	Age (n,%)*	Gender (n,%)*	Race/ Insurance (n,%)*
GPT-4o mini	131	18.3%	Age / Gender	84 (64.1%)	84 (64.1%)	21 (16.0%)
Claude 3.5 Sonnet	101	28.7%	Gender	42 (41.6%)	73 (72.3%)	15 (14.9%)
Grok	85	48.2%	Age	58 (68.2%)	53 (62.4%)	9 (10.6%)
Overall (Total)	317	29.7%	Gender	184 (58.0%)	210 (66.2%)	45 (14.2%)

* Note: Metadata distractors were not mutually exclusive; a single MIPD case could be triggered by multiple demographic fields (e.g., both Age and Gender). Percentages for distractors are calculated relative to the MIPD count of each row.

MIPD is defined as instances where a model provided a correct interpretation (Score 3) without metadata but produced a sub-optimal response (Scores 1 or 2) after the inclusion of patient context. Incidence rates are calculated relative to 6,000 paired no-metadata/with-metadata comparisons (2,000 per generator). The 'Hallucination in MIPD' column represents the proportion of degradation cases that specifically involved the generation of unsupported clinical findings. The 'Primary Metadata Distractor' indicates the demographic field most frequently associated with performance reversal.

Note S4. Additional Results of Experts Evaluation

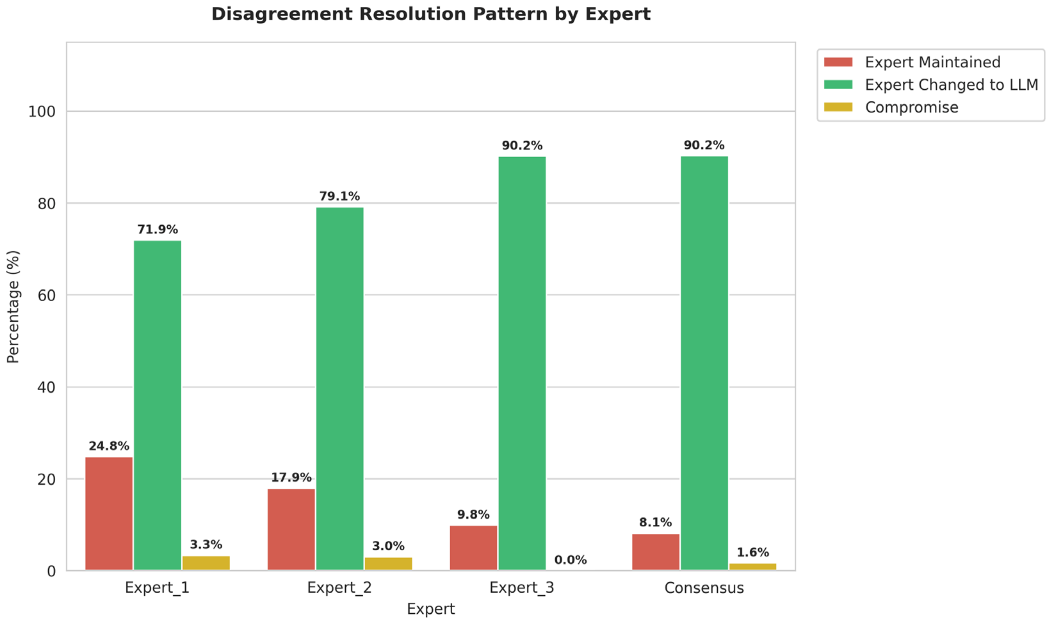

Figure S10. Resolution patterns of disagreements between human experts and the LLM.

Bar chart illustrating how initial discrepancies between expert and LLM scores were resolved during the final evaluation phase. "Expert revised after reviewing LLM-generated rationale" indicates cases where the expert final consensus matched the LLM judge. "Expert Maintained" indicates cases where the expert adhered to their initial score despite the LLM's suggestion. "Compromise" represents cases where the final score differed from both the initial and LLM scores. The final expert consensus matched the LLM judge in 90.2% of conflict cases.

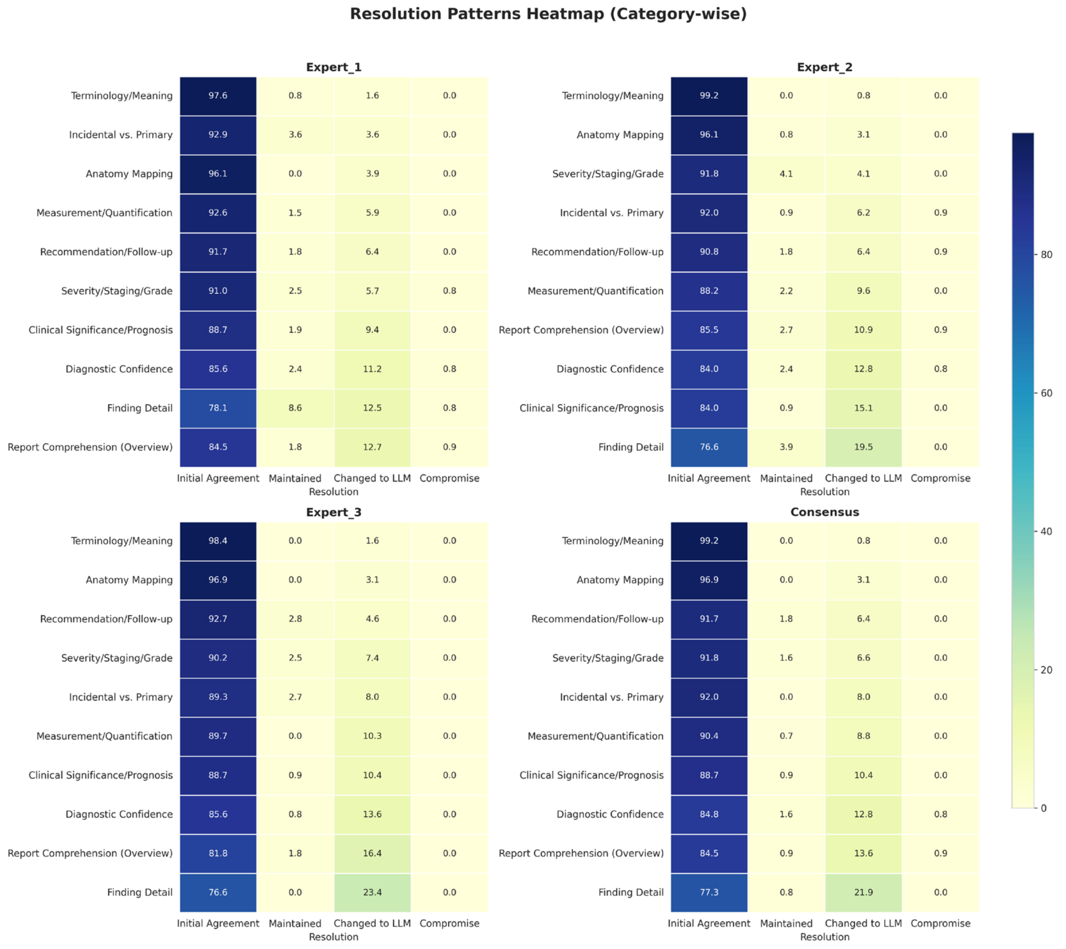

Figure S11. Heatmap comparison of resolution patterns per clinical category.

The 2x2 panel displays heatmaps showing the percentage of cases for each resolution type (Initial Agreement, Maintained, revised after reviewing LLM-generated rationale, Compromise) across all clinical categories. Darker shades indicate higher percentages. While the *Terminology/Meaning* category showed the highest initial agreement (>97%), categories requiring complex clinical judgment, such as *Recommendation/Follow-up* and *Anatomy Mapping*, showed a relatively higher proportion of experts revised after reviewing LLM-generated rationale.

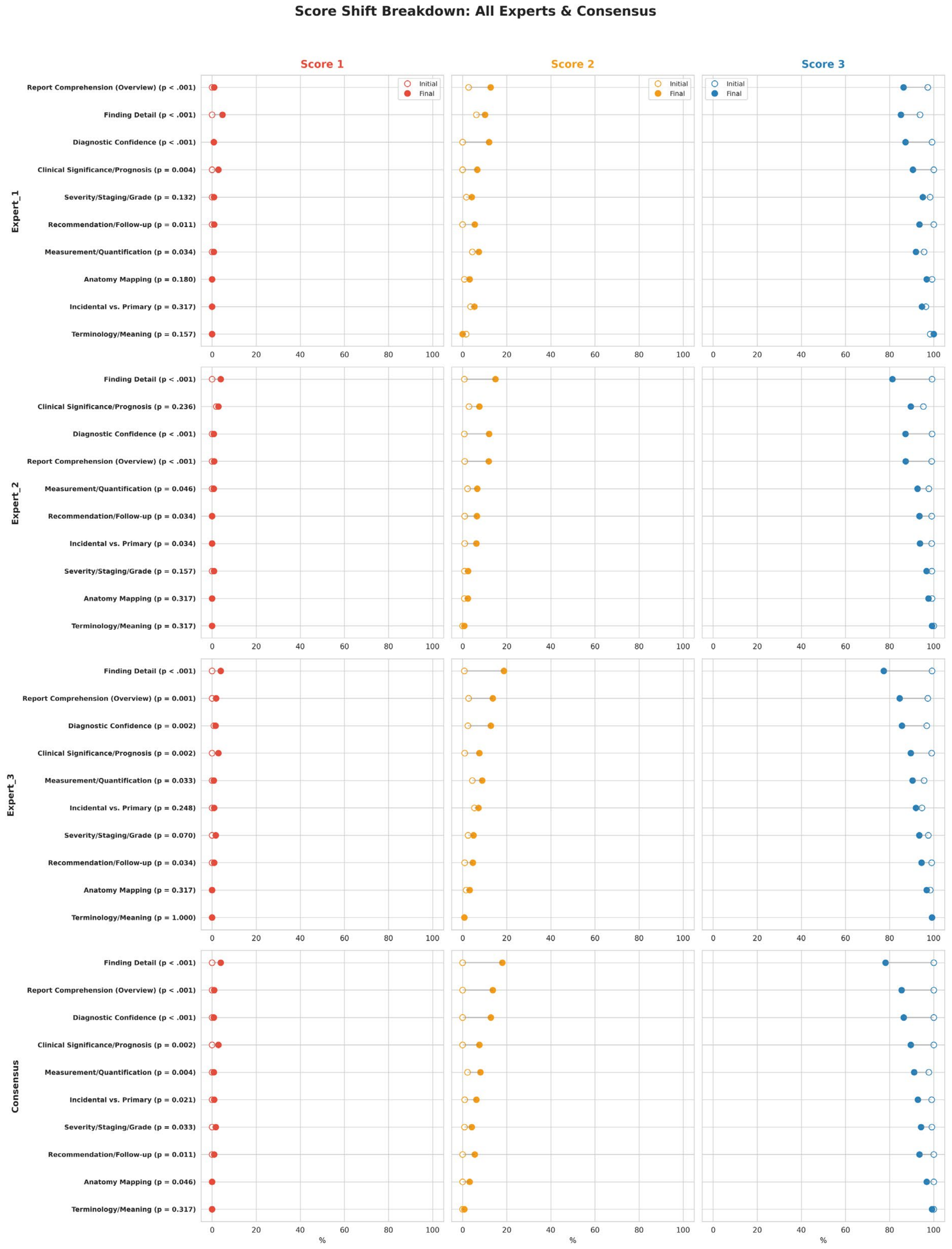

Figure S12. Category-wise shifts in quality score distributions before and after LLM review.

Multi-panel scatter plots demonstrating the change in the proportion of quality scores—Score 1 (Significant Error, left column, red), Score 2 (Minor Error, middle column, yellow), and Score 3 (Accurate, right column, blue)—for each clinical category. Rows correspond to the evaluators (Expert 1–3 and Consensus). Hollow circles represent the initial percentage, and solid circles represent the final percentage after LLM review. Horizontal lines indicate the magnitude of the shift. Statistical significance of the distribution shift was assessed using the Wilcoxon signed-rank test; *p* values are provided next to category labels. Significant improvements (increases in Score 3 and decreases in Score 1/2) were observed in the *Recommendation/Follow-up* (*p* =0.011), *Incidental vs. Primary* (*p* =0.021), and *Severity/Staging/Grade* (*p* =0.033) categories within the consensus data.

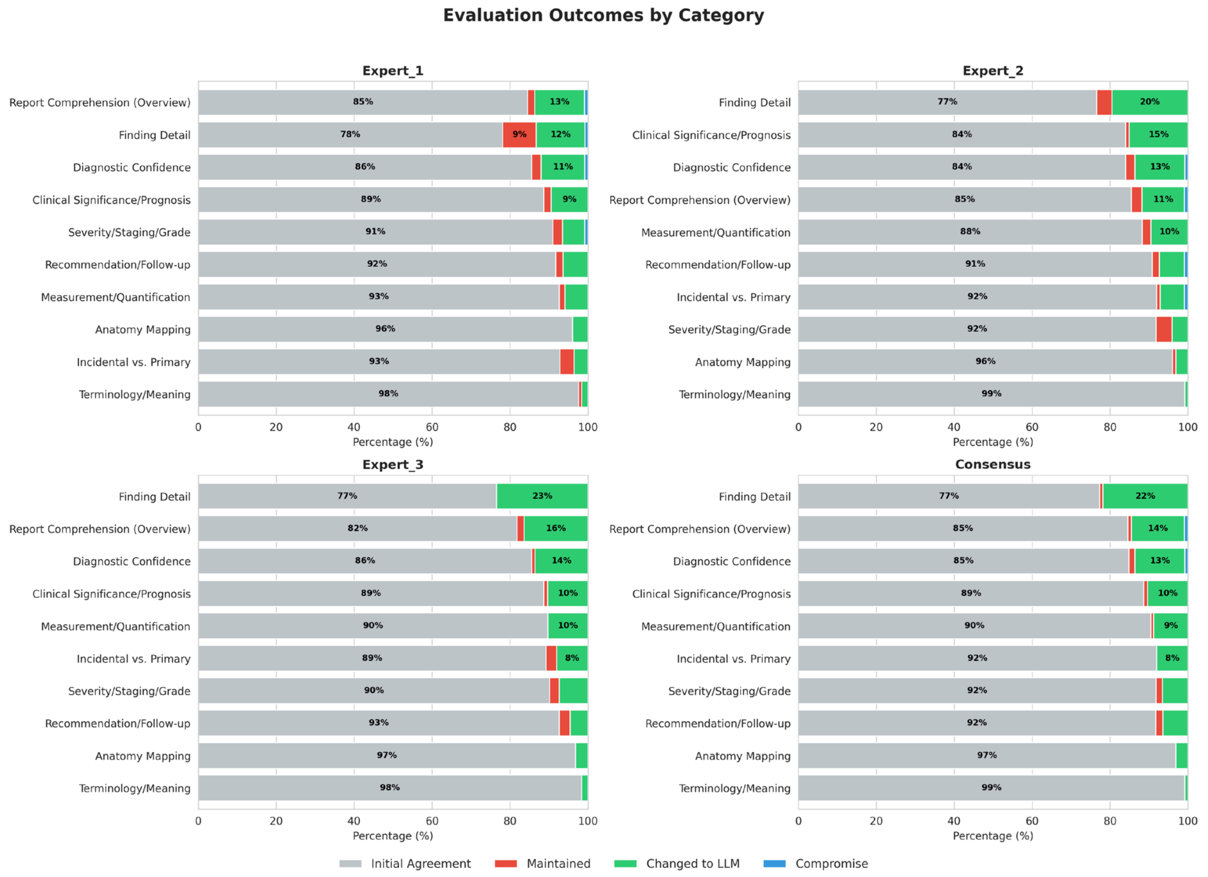

Figure S13. Distribution of evaluation outcomes by clinical category across individual experts and consensus.

Stacked bar charts illustrate the breakdown of final decision types for each clinical category across three experts (Expert 1, Expert 2, Expert 3) and their consensus. The categories are sorted by the correction rate. "Initial Agreement" (gray) represents cases where the expert's initial score matched the Large Language Model (LLM) score. " revised after reviewing LLM-generated rationale " (green) indicates cases where the expert final consensus matched the LLM judge. "Maintained" (red) denotes cases where the expert adhered to their initial score despite disagreement. "Compromise" (blue) refers to cases where the final score differed from both the initial and LLM scores. The highest rates of score modification ("revised after reviewing LLM-generated rationale ") were observed in the *Incidental vs. Primary* and *Severity/Staging/Grade* categories.

Table S6. Qualitative Analysis of LLM-Generated Radiology Interpretations: Representative Cases of Performance Heterogeneity, Metadata Bias, and Consensus Harmonization.*.

Case_Id & Note_Id	Case_Type	Gen_LLM (Eval_ Score by Gemini)	Clinical Context (Report & Metadata)	Question Category & Dynamic Question	LLM Answer & Clinical Critique
**Case1 (10038606-RR-6)**	**Performance Heterogeneity (Factual Mastery)**	**GPT-4o mini (3)**	**[Original Report]**<br>AP CHEST 5:45 P.M. ON ___<br>HISTORY: ___ woman with ischemic bowel. Right IJ central venous line placed and ET tube pulled back.<br>IMPRESSION: AP chest compared to ___ at 2:11 p.m. Endotracheal tube has been pulled back from the carina to a standard placement in the trachea. There is probably no appreciable atelectasis or other focal pulmonary abnormality. Mediastinal contours are distorted by patient rotation, but the thoracic aorta appears to be generally very large, tortuous, and <u>may have a focal aneurysm</u> <u>extending from the level of the left hilus to the diaphragm</u>, or a hiatus hernia simulating an aneurysm. Heart is only top normal size. There is no pulmonary edema. Tip of the new right jugular line projects over the superior cavoatrial junction. Nasogastric tube ends above the diaphragm either in the esophagus parallel to the tortuous aorta, or in the intrathoracic stomach. No pneumothorax, mediastinal widening or pleural<br>effusion. Dr. ___ and I discussed these findings at the time of dictation.<br><br>**[Metadata]**<br>FALSE	**TERMINOLOGY/MEANING:**<br>*"I don't understand the word 'aneurysm'. What does it mean?"*	**[LLM Answer]**<br><u>An aneurysm is a bulge or swelling in a blood vessel caused by weakness in the vessel wall.</u> In your report, it suggests that there <u>may be a bulge in the thoracic aorta</u>, which is the large artery that carries blood from the heart to the rest of the body. It's important to monitor this condition, as it can lead to serious health issues if it grows or ruptures.<br><br>**[Clinical Critique]**<br>Factual Mastery.<br>The LLM demonstrated high precision in medical terminology retrieval, functioning as a reliable dictionary for patients.
**Case2 (10038606-RR-6)**	**Performance Heterogeneity (Interpretive Failure)**	**GPT-4o mini (1)**	**[Report] and [Metadata]**<br>(Same as Above)	**FINDING DETAIL:**<br>*"Where exactly is the focal aneurysm and how big is it?"*	**[LLM Answer]**<br>The 'focal aneurysm' mentioned in your report is likely located in the thoracic aorta, which is the large blood vessel that carries blood from your heart to the rest of your body. The report suggests that this aneurysm may extend from the area near your left lung (left hilus) down to the diaphragm. <u>Unfortunately, the report does not specify the exact size of the aneurysm or provide a visual</u>

					description, but it indicates that the aorta appears very large and tortuous. It's important to discuss these findings with your doctor for more personalized information and next steps.<br><br>**[Clinical Critique]**<br>Inference Gap.<br>The AI failed to synthesize specific descriptors (e.g., "left hilus to diaphragm") into an actionable answer. This reasoning failure can mislead patients about the specificity of their findings.
**Case3 (10248241-RR-30)**	**Score Spectrum (Accurate and Comprehensive)**	**Grok (3)**	**[Report]**<br>EXAMINATION: MRI of the Abdomen<br>INDICATION: ___ year old man with mixed HCC/cholangio, s/p TACE x2 and RFA.<br>Assess for interval change at 1 month<br>TECHNIQUE: T1- and T2-weighted multiplanar images of the abdomen were<br>acquired in a 1.5 T magnet.<br>Intravenous contrast: 9 mL Gadavist.<br>COMPARISON: CT interventional procedure ___<br>MRI liver ___.<br>FINDINGS:<br>Lower Thorax: Limited evaluation of the lung bases are clear. No pleural effusion.<br>Liver: Liver is nodular in contour with heterogeneous reticular enhancement consistent with cirrhosis and fibrosis. Status post radiofrequency ablation and TACE of segment 6 (most recent RFA on ___ with 6.1 x 5 cm treatment cavity with intrinsic T1 hyperintensity consistent with coagulation necrosis. No nodular enhancement to suggest residual or recurrent disease (10:39). Geographic peripherally located area of arterial hyper enhancement within segment 6 and 7 is without correlate on additional sequences and consistent with transient hepatic intensity differences (10:41,16).	**REPORT COMPREHENSION (OVERVIEW):**<br>"*Can you summarize the overall findings in simple words?*"	**[LLM Answer]**<br>Sure, I'll summarize the key findings from your abdominal MRI in simple terms. Your liver shows signs of cirrhosis (scarring) and an enlarged spleen, which is common with that. You've had treatments for liver cancer (like ablation and embolization), and the treated area in segment 6 looks good with no signs of the cancer returning or leftover issues. There's a small, unchanged spot in segment 2 of the liver that's not specific but stable-we'll keep an eye on it. No new concerning liver spots that suggest cancer. There are two new small blood clots in branches of the liver veins. In your pancreas, there's a 2 cm cyst in the head that's the same size as before and likely a benign type called IPMN; we'll monitor that too. You have gallstones, but no signs of gallbladder inflammation or infection. Your kidneys have some simple cysts, but nothing worrisome. Everything else, like your lungs, adrenal glands, bowels, and blood vessels, looks normal with no major problems. No fluid buildup in your belly. The doctor recommends follow-up scans to watch the liver spot and pancreas cyst.<br><br>**[Clinical Critique]**<br>High-Fidelity Synthesis.

	Additional rounded 0.9 cm arterially hyperenhancing lesion within segment 2 is not seen on additional sequences and is unchanged since prior examination (10:14). No signal drop on out of phase imaging to suggest hepatic steatosis.<br>No ascites.<br>Biliary: No intrahepatic or extrahepatic biliary duct dilatation.<br>Cholelithiasis noted without gallbladder distention or gallbladder wall edema.<br>Pancreas: Again seen is a 1.9 x 2.1 x 1.6 cm (previously 1.9 x 2.1 x 1.6 cm) multilobulated T2 hyperintense cystic lesion within the pancreatic head. The pancreas is otherwise homogeneous in signal intensity. No pancreatic duct dilatation. No peripancreatic fat stranding.<br>Spleen: Enlarged measuring 15.7 cm without focal lesion.<br>Adrenal Glands: Unremarkable.<br>Kidneys: Bilateral T2 hyperintense nonenhancing renal cysts are noted. No<br>hydronephrosis. No perinephric fat stranding.<br>Gastrointestinal Tract: Distal esophagus, stomach, visualized small and large<br>bowel are unremarkable. No obstruction.<br>Lymph Nodes: No retroperitoneal or mesenteric lymph node enlargement.<br>Vasculature: No abdominal aortic aneurysm. Celiac axis, SMA, and bilateral renal arteries are patent. Early origin of the right hepatic artery from the common hepatic artery. Hepatic veins, main portal vein, SMV, and splenic vein are patent. Two new linear tracts extending from the ablation cavity are consistent with segmental right hepatic vein thrombus (12:42).<br>Osseous and Soft Tissue Structures: Unremarkable.<br>IMPRESSION:<br>1. Status post hepatic segment 6 radiofrequency ablation with expected	Grok successfully prioritized clinical urgency by capturing the "new thrombus"—the most critical interval change. It distinguishes acute risks from stable findings, supporting safer interpretation in this case.

			coagulation necrosis. No abnormal enhancement to suggest residual or recurrent disease.<br>2. Non-specific 0.9 cm enhancing segment 2 lesion is unchanged.<br>3. No worrisome hepatic lesions meeting OPTN 5 criteria for HCC.<br>4. Two new thrombosed segmental right hepatic vein branches.<br>5. Cirrhosis with splenomegaly. No ascites.<br>6. 2.1 cm pancreatic head cystic lesion, unchanged since ___, likely IPMN.<br>7. Cholelithiasis without evidence of acute cholecystitis.<br>RECOMMENDATION(S): Close attention on follow-up is recommended of segment 2 hepatic lesion and pancreatic head cystic lesion.<br><br>**[Metadata]**<br>TRUE(Age, Gender)		
**Case4 (10248241-RR-30)**	**Score Spectrum (Ambiguous or Incomplete)**	**GPT-4o mini (2)**	**[Report] and [Metadata]**<br>(Same as Above)	(Same as Above)	**[LLM Answer]**<br>The MRI shows that your liver has some scarring and changes from previous treatments, <u>but there are no signs of new cancer.</u> There is a small cyst in your pancreas that hasn't changed, and your spleen is larger than normal. You also have gallstones, but there are no signs of infection. It's important to keep an eye on the small liver lesion and the pancreatic cyst in future check-ups.<br><br>**[Clinical Critique]**<br>Critical Omission. The AI provided a linguistically fluent but "Safe-but-Incomplete" summary. By omitting acute findings, it leaves the patient unaware of new clinical threats that require immediate attention.
**Case5 (10248241-RR-30)**	**Score Spectrum (Inaccurate or Clinically significant Error)**	**Claude 3.5 Sonnet (1)**	**[Report] and [Metadata]**<br>(Same as Above)	(Same as Above)	**[LLM Answer]**<br>The MRI shows that your liver has some scarring (cirrhosis) and you've had previous treatments for liver cancer. The good news is that there's no sign of the cancer coming back in the treated area. There's a small spot in another part of your liver that

					hasn't changed, which is <u>reassuring</u>. You have some gallstones, but they're <u>not causing problems right now</u>. There's also a small cyst in your pancreas that hasn't changed. Your spleen is larger than normal, which can happen with liver disease. The doctors recommend keeping a close eye on the small liver spot and the pancreas cyst in future check-ups.<br><br>**[Clinical Critique]**<br>False Reassurance. Beyond omission, the model inappropriately downplayed clinical severity. Calibration Deficit. A high-confidence "silent failure" that could delay appropriate clinical follow-up in a real-world context.
**Case6 (11151057-RR-11)**	**Metadata Paradox (Without Metadata)**	**Grok (3)**	**[Report]**<br>EXAMINATION: CHEST (PA AND LAT)<br>INDICATION: ___ year old woman s/p fall, ? R ___/ 4th rib fx and hemothorax<br>// eval for ? evolution of effusion<br>COMPARISON: ___<br>IMPRESSION:<br><u>No relevant change</u> as compared to the previous image. No development of larger pleural effusions. No effusions are seen on the lateral radiograph. The pre-existing parenchymal and cardiac changes as well as the elongation of<br>the descending aorta are constant..<br><br>**[Metadata]**<br>FALSE	**DIAGNOSTIC CONFIDENCE:**<br>"*Where exactly is the '4th rib fx' in my body, how big is it, and what does it look like?*"	**[LLM Answer]**<br>Based on the radiology report provided, <u>there is no mention or confirmation of a 4th rib fracture.</u> The report does not describe any specific rib fractures or their appearance. Instead, it states that there are no significant changes compared to a previous image and no development of larger pleural effusions (fluid around the lungs). The report focuses on the stability of pre-existing conditions rather than describing any new or specific rib injuries.<br><br>**[Clinical Critique]**<br>The LLM demonstrated high fidelity to the formal report structure by correctly distinguishing between the 'Indication' (clinical suspicion) and the 'Impression' (radiographic confirmation). Unlike the metadata-inclusive version, the model remained strictly objective, noting that the suspected fracture was not specifically confirmed in the impression. By acknowledging the diagnostic limitations of a standard chest X-ray for rib fractures and deferring to professional clinical

					correlation, the AI avoided a false-positive hallucination. This response serves as a benchmark for 'Defensive AI Reasoning,' prioritizing documented evidence over statistical probability.
**Case7 (11151057-RR-11)**	**Metadata Paradox (With Metadata)**	**Grok (1)**	**[Report]**<br>(Same as Above)<br><br>**[Metadata]**<br>TRUE(Age, Gender, Insurance)	(Same as Above)	**[LLM Answer]**<br>The radiology report notes a suspected right 4th rib fracture (often abbreviated as 'rib fx') related to your recent fall, along with a check for possible hemothorax (blood in the chest cavity) and any changes in fluid buildup.<br>The good news is that this follow-up chest X-ray shows no significant changes compared to your previous scan — no new or worsening fluid collections (effusions), and stable findings in your lungs, heart, and aorta. This suggests things are not getting worse.<br>For a rib fracture like this, <u>especially at age 87</u>, the next steps usually focus on healing and symptom management rather than surgery, as most rib fractures heal on their own over 4-6 weeks. Here's what you can do: 1.Rest and avoid activities that strain your chest, like heavy lifting or intense exercise. 2.Manage pain with over-the-counter options like acetaminophen (Tylenol) or ibuprofen (Advil), but check with your doctor first, especially if you have other health conditions or take medications. Ice packs can help with swelling. 3. Practice deep breathing exercises to prevent complications like pneumonia — try taking slow, deep breaths or using an incentive spirometer if prescribed. 4.Follow up with your primary care doctor or the physician who ordered the X-ray for personalized guidance, possibly including a repeat scan in a few weeks to confirm healing. <u>If you have Medicare, this should cover follow-up visits.</u>

					Watch for warning signs like increasing chest pain, shortness of breath, coughing up blood, or fever, and seek immediate medical help if they occur — call 911 or go to the ER. Remember, I'm providing general information based on the report; your doctor knows your full medical history and can give the best advice.<br><br>**[Clinical Critique]**<br>While the LLM correctly identified the absence of a fracture in the impression without metadata, the introduction of patient descriptors triggered a critical hallucination. The model explicitly verbalized the metadata—"especially at age 87" and "If you have Medicare"—and prioritized demographic statistical likelihoods over the objective radiographic findings. By prescribing management for a non-existent fracture, the AI demonstrated a 'Silent Failure' where linguistic fluency masks a profound clinical error induced by demographic priors.
**Case8 (14841375-RR-13)**	**Expert Convergence (Harmonizer)**	**GPT-4o mini (1)**	**[Report]**<br>INDICATION: ___ woman with right hip pain following MVC.<br>COMPARISON: None.<br>SINGLE SUPINE VIEW OF THE CHEST AT 5:30 P.M.: Evaluation is limited by<br>overlying trauma board. Lung volumes are low, but there is no focal consolidation, pleural effusion or pneumothorax. The cardiomediastinal<br>silhouette demonstrates relative mediastinal widening, likely due to low lung<br>volumes and technique. Rib fractures are noted of the left lateral fourth, fifth, sixth and seventh ribs. No other fractures are identified.<br>SINGLE AP VIEW OF THE PELVIS: Evaluation is limited by trauma board and	**REPORT COMPREHENSION (OVERVIEW):**<br>"*I don't really understand. Can you summarize the overall findings in simple words*?"	**[LLM Answer]**<br>The report shows that you have some broken ribs on the left side and a dislocated right hip, but there are no major fractures in the hip. Your lung volume is low, and there are signs of wear and tear in your lower back. Overall, while there are some injuries, there are no serious fractures detected.<br><br>**[Clinical Critique]**<br>Consensus Harmonizer. Safety-driven Consensus Harmonization: The initial expert disagreement ([2, 3, 3]) was resolved through the LLM's logical evidence-tracing, which exposed a critical safety oversight. While the response appeared fluent, its conclusion—'no serious fractures detected'—directly contradicted the presence of four acute rib fractures and a major hip

	patient positioning. However, there is superolateral dislocation of the right femoral head relative to the acetabulum. The femoral neck appears intact, but<br>visualization is limited. Severe degenerative changes are noted in the lumbar<br>spine. No other acute abnormalities are present.<br>IMPRESSIONS:<br>1.Fractures through lateral left fourth through seventh ribs.<br>2.Right hip dislocation, without displaced fracture, although evaluation for<br>fracture is limited.<br>3.Low lung volumes.<br>4.Degenerative changes in the lower lumbar spine.<br><br>**[Metadata]**<br>TRUE(Age, Gender, Race, Insurance)		dislocation. This case illustrates how the LLM-aided audit helps standardize safety evaluations by detecting clinically misleading summaries that individual human reviewers might initially overlook. Experts scores changed from [2,3,3] to [1,1,1]

* This table presents representative clinical cases illustrating the qualitative performance of Large Language Models (LLMs) across five distinct evaluative scenarios. Case_id & Note_Id refers to table raw index and the unique identifier from the MIMIC-IV radiology dataset. Case_Type categorizes the specific failure or success mode observed: Performance Heterogeneity highlights the discrepancy between factual knowledge and interpretive reasoning; Score Spectrum demonstrates inter-model variability in clinical prioritization; Metadata Paradox illustrates performance reversal triggered by demographic priors; and Expert Convergence showcases the LLM's role as a consensus harmonizer. Clinical Context (Report & Metadata): Excerpts from the original radiology report and the specified prompting condition. TRUE indicates that patient demographic metadata (e.g., age, gender) was provided to the LLM as a context enhancer, while FALSE indicates a report-only prompting condition. Question Category & Dynamic Question: The functional classification of the inquiry and the specific simulated patient question. LLM Answer & Clinical Critique: The raw LLM output and the subsequent medical audit.

End of Document
